# The ant genus *Carebara* Westwood (Hymenoptera, Formicidae): synonymisation of *Pheidologeton* Mayr under *Carebara*, establishment and revision of the *C. polita* species group

**DOI:** 10.3897/zookeys.438.7922

**Published:** 2014-09-01

**Authors:** Georg Fischer, Frank Azorsa, Brian L. Fisher

**Affiliations:** 1 Entomology, California Academy of Sciences, 55 Music Concourse Drive, San Francisco, CA 94118, U.S.A.; 2 División de Entomologia, Centro de Ecologia y Biodiversidad, CEBIO. Lima, PERU; 3 Department of Biological Sciences, San Francisco State University, 1600 Holloway Ave. San Francisco, CA 94132, U.S.A.

**Keywords:** *Carebara*, *Pheidologeton*, new genus synonymy, *Oligomyrmex*, *Paedalgus*, taxonomic revision

## Abstract

In this paper the genus *Pheidologeton* Mayr, 1862 is synonymized under *Carebara* Westwood, 1840 and the *Carebara polita* group is established and revised. This species group currently includes six species from the Afrotropical region (*C. madibai*, *C. nicotianae*, *C. perpusilla*, *C. polita*, *C. silvestrii*, and *C. villiersi*) and two species from the Neotropical region (*C. brevipilosa* and *C. urichi*). The *polita* group clearly links *Carebara* and *Pheidologeton*, and, due to a lack of autapomorphic characters for the latter, a separation of the two genera is no longer justified. As a result *Carebara* is presented as a monophyletic and better defined genus that can be separated from other genera with more confidence. We present an overview of the distribution and biology of *Carebara* as well as images from the various genera currently in synonymy under *Carebara*, and discuss the characters they share. The polymorphism present in Afrotropical and Malagasy *Carebara* is discussed and one new species from Africa, *C. madibai* **sp. n.**, is described. The subspecies *Carebara perpusilla arnoldiana* **syn. n.**, *Carebara perpusilla concedens* **syn. n.**, and *Carebara perpusilla spinosa* **syn. n.** are new synonyms of *Carebara perpusilla*. *Oligomyrmex politus nicotianae* is re-elevated to species level and transferred into *Carebara*, *C. nicotianae* **comb. n.**, **stat. rev.**; *C. punctata* is a new synonym of *C. silvestrii* **comb. n.** and *C. pygmaea albipes* **comb. n.**, **syn n.**, *C. pygmaea bugnioni* **comb. n.**, **syn. n.**, and *C. simularensis* **syn. n.** are new synonyms of *C. pygmaea* **comb. n..** The following names are transferred from *Pheidologeton* to *Carebara* as new combinations (with the species epithets adjusted to female endings where necessary): *aberrans*, *affinis*, *affinis javana*, *affinis minor*, *affinis spinosior*, *affinis sumatrensis*, *ceylonensis*, *dentiviris*, *diversa*, *diversa draco*, *diversa ficta*, *diversa laotina*, *diversa macgregori*, *diversa philippina*, *diversa standfussi*, *diversa taprobanae*, *diversa tenuirugosa*, *diversa williamsi*, *hammoniae*, *hostilis*, *kunensis*, *latinoda*, *maccus*, *mayri*, *melanocephala*, *melasolena*, *nana*, *nanningensis*, *obscura*, *petulens*, *pullata*, *pungens*, *pygmaea*, *rubra*, *rugiceps*, *rugosa*, *schossnicensis*, *silena*, *silvestrii*, *solitaria*, *transversalis*, *trechideros*, *varia*, *vespilla*, *volsellata*, *yanoi*, and *zengchengensis*. Three new combinations are creating secondary junior homonyms and are here replaced with new names: *C. mayri* (Santschi, 1928) = *C. gustavmayri* **nom. n.**, *C. rugosa* (Karavaiev, 1935) = *C. rugoflabella* **nom. n.**, and *C. silvestrii* (Wheeler, 1929b) = *C. luzonensis* **nom. n.** Two new combinations are creating secondary junior homonyms among species already in Carebara: *C. taprobanae* (Forel, 1911a) = *C. sinhala* **nom. n.**, and *C. nana* Santschi, 1919 = *C. pumilia* **nom. n.**

## Introduction

The ant genus *Carebara* currently contains approximately 250 described taxa (including the newly described species and all taxonomic changes in this work), and is distributed worldwide, being present mainly in tropical and subtropical regions (Azorsa and Fisher, in review, Bharti and Kumar 2013, Bolton 2014, Sharaf and Aldawood 2013). Many undetermined specimens and morphospecies are present in museum collections around the world and with ongoing taxonomic research the species number within the genus is expected to grow considerably (FA and GF, unpublished data). *Carebara* contains some of the smallest (e.g. *Carebara minuta*) and largest (e.g. *Carebara aberrans* comb. n.) ants in the world. Relatively little is known about the biology of this genus, except for the generalized foraging and mass raiding habits of several marauder ant species (*Pheidologeton*: Moffett 1988, Berghoff et al. 2003). The majority of species are very small and seem to have cryptic lifestyles. Stable isotope analyses of six *Carebara* species from Kenya revealed predatory to specialized predatory diets (Fischer 2012).

In recent years, the boundaries of the ant genus *Carebara* have changed significantly, mainly because of a lack of a clear genus definition. The latter is a result of not only the incomplete species record, but also from taxonomic difficulties inherent in a group that contains many cryptic and morphologically reduced species and that is also highly diverse, both morphologially and ecologically. These boundaries have changed constantly, especially with the extensive treatments by Ettershank (1966) and Fernández (2004, 2010). Ettershank (1966) split the tribes Solenopsidini and Pheidologetini into four genus groups, *Monomorium*, *Megalomyrmex*, *Pheidologeton* and *Solenopsis* group, and expanded the boundaries of the genus *Oligomyrmex*, (included in the *Pheidologeton* genus group), by synonymizing *Aeromyrma*, *Pheidologeton* subgenera *Aneleus* & *Lecanomyrma*, *Erebomyrma*, *Oligomyrmex* subgenera *Octella* & *Hendecatella*, *Spelaeomyrmex*, *Solenopsis* subgenera *Solenops* & *Crateropsis*, and *Nimbamyrma* under *Oligomyrmex*. Bolton (2003) included Pheidologetini in Solenopsidini and split all the genera into the *Carebara* and *Solenopsis* groups. The *Carebara* group was represented by the genera *Adlerzia*, *Afroxyidris*, *Carebara*, *Machomyrma*, *Mayriella*, *Oligomyrmex*, *Paedalgus*, *Pheidologeton* and *Tranopelta*. Bolton and Belshaw (1993), and Belshaw and Bolton (1994) pointed out that *Afroxyidris* and *Paedalgus* are closely related to *Carebara* and *Oligomyrmex*. Fernández (2004), in the revision of the American fauna expanded the boundaries of *Carebara*, by including *Afroxyidris*, *Oligomyrmex* and *Paedalgus*.

Fernández (2010) included *Parvimyrma* in *Carebara* and also suggested the inclusion of *Pheidologeton* as a junior synonym of *Carebara*, mainly because of the morphological similarity between *Carebara villiersi* and *Pheidologeton* workers, and the similarity of the sting apparatus of *Carebara*, *Oligomyrmex* and *Pheidologeton* (Kugler 1986). Despite Fernández’s suggestion *Pheidologeton* continued to be recognized as a separate genus mainly because its worker caste is polymorphic, often with continuous series of intermediates between minor and large major workers, versus a monomorphic and dimorphic worker caste in *Carebara*. However, in a recent study of *Carebara* from the Malagasy Region, Azorsa and Fisher (in review) found that most *Carebara* species were polymorphic, with one to four intermediate subcastes being present between the small and large major workers. These findings (and unpublished data on polymorphic *Carebara* species from Africa with a specialized phragmotic major worker subcaste) suggest that a pronounced worker polymorphism could also be present in other species of *Carebara*. Given this morphological evidence we consider *Pheidologeton* likely nested within *Carebara* and thus not deserving generic status.

The differences between intermediate worker subcastes in *Carebara* are mainly as follows: head and mesosoma size, size of posterolateral head corners, eye size, number of ocelli from one to three (ocelli are reduced compared to the queen caste), and full-sized versus reduced flight sclerites. Intermediates or additional major worker subcastes are also present in other groups of ants (e.g. *Acanthomyrmex*, *Camponotus*, *Cephalotes*, *Crematogaster*, *Pheidole*), and it is possible that the intermediates in *Carebara* are trophic specialists as in some of those genera (see Molet et al. 2012, and Peeters et al. 2013). Studies of some *Carebara* species (*Carebara overbecki*, *Carebara urichi*) found that the colonies contain up to 1000 individuals (minor and major workers) and that the proportion of the major workers in one nest can approach ten percent (Moffett 1986, Wilson 1962). Smaller workers nurse the brood, and larger workers defend the nest. The diets of these ants include mites, entomobryid collembolans and arthropod eggs (Wilson 1962, 1986).

In addition to the inclusion of *Pheidologeton* within *Carebara*, we establish and revise the *Carebara polita* species group, which is morphologically very close to *Pheidologeton*, with eight species from the Afrotropical and Neotropical regions. We also provide high-quality montage images from a few typical morphologies of genera synonymised under *Carebara* and discuss the similarities between them.

### Abbreviations of depositories

BMNH The Natural History Museum (British Museum, Natural History), London, U.K.

CASC California Academy of Sciences, San Francisco, CA, U.S.A.

IAvH Insect Collection, Instituto Humboldt, Claustro de San Agustín, Villa de Leyva, Colombia

LACM Los Angeles County Museum of Natural History, Los Angeles, CA, U.S.A.

MCZ Museum of Comparative Zoology, Cambridge, Massachusetts, U.S.A.

MHNG Muséum d’Histoire Naturelle de la Ville de Genève, Geneva, Switzerland

MNHN Muséum National d’Histoire Naturelle, Paris, France

MSNG Museo Civico di Storia Naturale “Giacomo Doria”, Genova, Italy

NHMB Naturhistorisches Museum, Basel, Switzerland

PSWC Philip S. Ward Collection, University of California, Davis, CA, U.S.A.

## Material and methods

The majority of specimens examined in this work are deposited in the ant collection at CASC, and PSWC. We also examined type material of *Carebara* and *Pheidologeton* deposited at MHNG. Specimens were examined using a Leica M165 (maximum magnification 160×).

For morphological characters we followed Bolton (1994), Bolton and Belshaw (1993), Belshaw and Bolton (1994), Ettershank (1966), Bolton (2003) and Fernández (2004, 2006, 2010). For sculpture characters we followed Harris (1979). The terminology we used to describe the inclination of pilosity follows the five types of hair inclination described by Wilson (1955). **Erect:** hairs that are vertical or nearly vertical to the cuticular surface (inclination 90°); **suberect:** hairs with an inclination of 70° from the cuticular surface; **subdecumbent:** hairs with an inclination of 50° from the cuticular surface; **decumbent:** hairs with an inclination of 30° from the cuticular surface; and **appressed:** hairs with an inclination of 10° or nearly parallel to the cuticular surface. The hairs described may be of different sizes. In most cases the larger hairs are usually two to three times the size of the shorter pilosity and have a different inclination than their smaller counterparts.

We present high-resolution images of all *polita* group species, as well as a comparison of some representatives of the genera previously synonimized under *Carebara* (Fig. 2). Images were created using a JVC KY-F75 digital camera and Syncroscopy Auto-Montage software (version 5.0), or a Leica DFC 425 camera in combination with the Leica Application Suite software (version 3.8). All images were edited using Photoshop and Adobe Ilustrator software. Images can be viewed and downloaded at www.AntWeb.org. Updatable distribution maps are also available via www.AntWeb.org. Distribution maps (Fig. 18) were generated with the freeware R (R Core Team 2014).

### Measurements and indices

All measurements were taken with a Leica M165 equipped with an orthogonal pair of micrometers up to a magnification of 160×.

**Figure 1.**
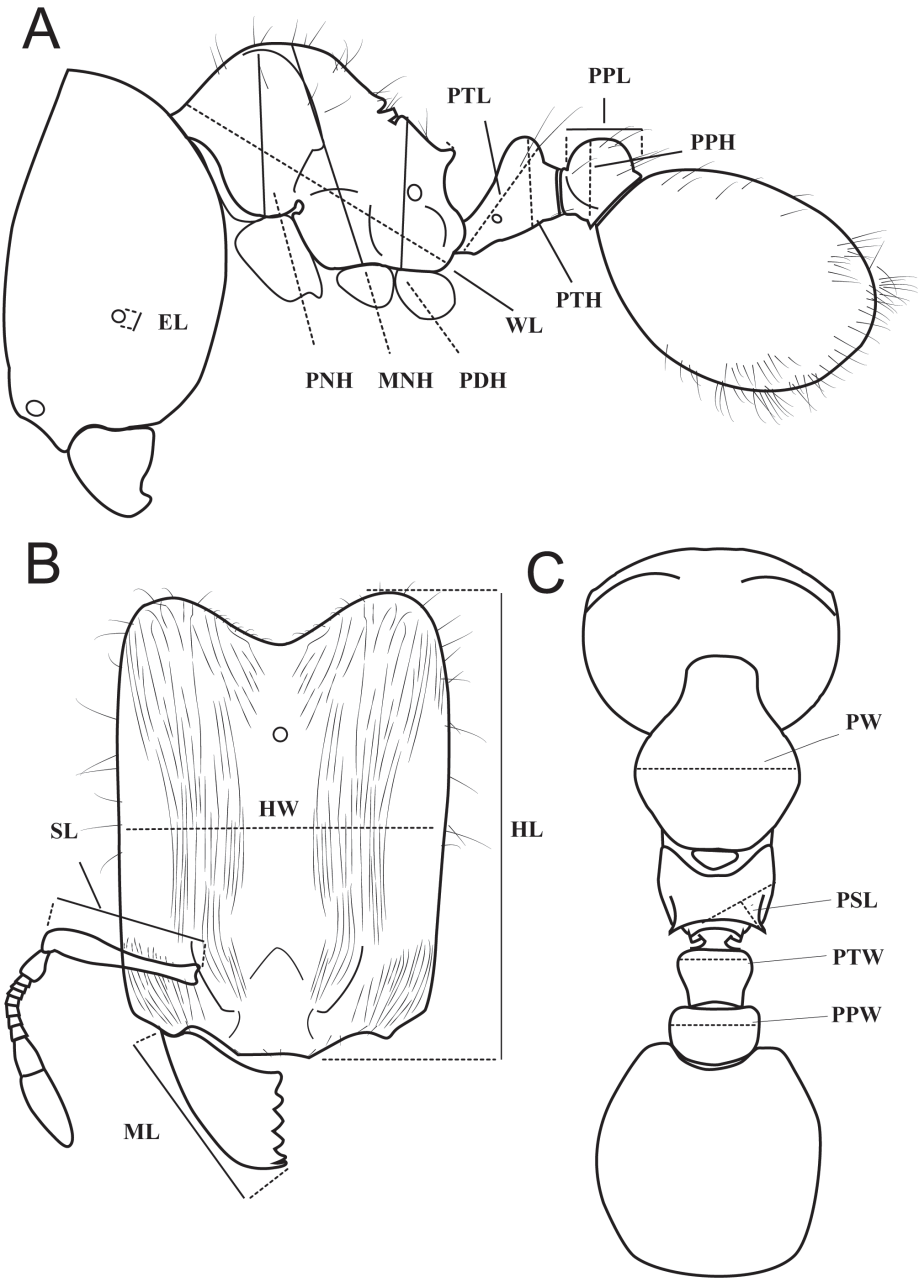
Illustration of standard measurements used for this revision: *Carebara* sp.: **A** profile **B** full-face view **C** dorsal view of major worker.

The following terminology and abbreviations are used (see Fig. 1):

HL head length: maximum distance from the midpoint of the anterior clypeal margin to the midpoint of the posterior margin of the head, measured in full-face view; in majors, measured from the midpoint between anteriormost positions of clypeus to midpoint between posteriormost projections of the posterolateral lobes.

HW head width: measured at widest point of the head in full-face view.

SL scape length: maximum scape length, excluding basal condyle and neck.

EL eye length: maximum diameter of compound eye measured in oblique lateral view.

MFL metafemur length: measured from the junction with the trochanter to the junction with the tibia.

MTL metatibia length: measured from the junction with femur to the junction with first tarsal segment.

MDL mandible length: maximum length, measured in oblique frontolateral view, from apex to lateral base.

PNW pronotal width: maximum width of pronotum measured in dorsal view.

WL Weber’s length: diagonal length of mesosoma in lateral view from the anterior point of the pronotal slope and excluding the neck, to the posteroventral margin of the propodeum.

PNH pronotum height: maximum height of pronotum, measured in profile from the posterior base of the lateral sides of pronotum, where procoxa is attached, to the highest point of the pronotum.

MNH promesonotum height: maximum height of promesonotum, measured in profile from the anterior base of the katepisternum, where mesocoxa is attached, to the highest point of the dorsal pronotum.

PDH propodeum height: maximum height of propodeum, measured in profile from the highest point of the dorsopropodeum perpendicular to a line that marks the lateroventral borders of the katepisternum and the propodeum.

PSL propodeal spine length: in dorsocaudal view, with the apex of the measured spine, its base, and the center of the propodeal concavity between the spines in focus: measurement is taken from apex to base along one axis of a dual–axis micrometer, which is aligned along the length of the spine, while the second axis crosses the base of the measured spine, and connects the base with the center of the propodeal concavity.

PTL petiole length: maximum diagonal length of petiole, measured in lateral view, from most anteroventral point of the peduncle, at or below the propodeal lobe, to most posterodorsal point at the junction withhelcial tergite.

PTH petiole height: maximum height of petiole, measured in lateral view from the highest (median) point of the node, orthogonally to the ventral outline of the node.

PTW petiole width: maximum width of the petiole node, measured in dorsal view.

PPL postpetiole length: maximum length of postpetiole, measured in lateral view, from anterior beginning of the dorsal slope to the posterior end of postpetiole tergite.

PPH postpetiole height: maximum height of postpetiole, measured in lateral view, from the highest (median) point of the node to the lowest point of the ventral process, often in an oblique line.

PPW postpetiole width: maximum width of postpetiole, measured in dorsal view.

**Figure 2.**
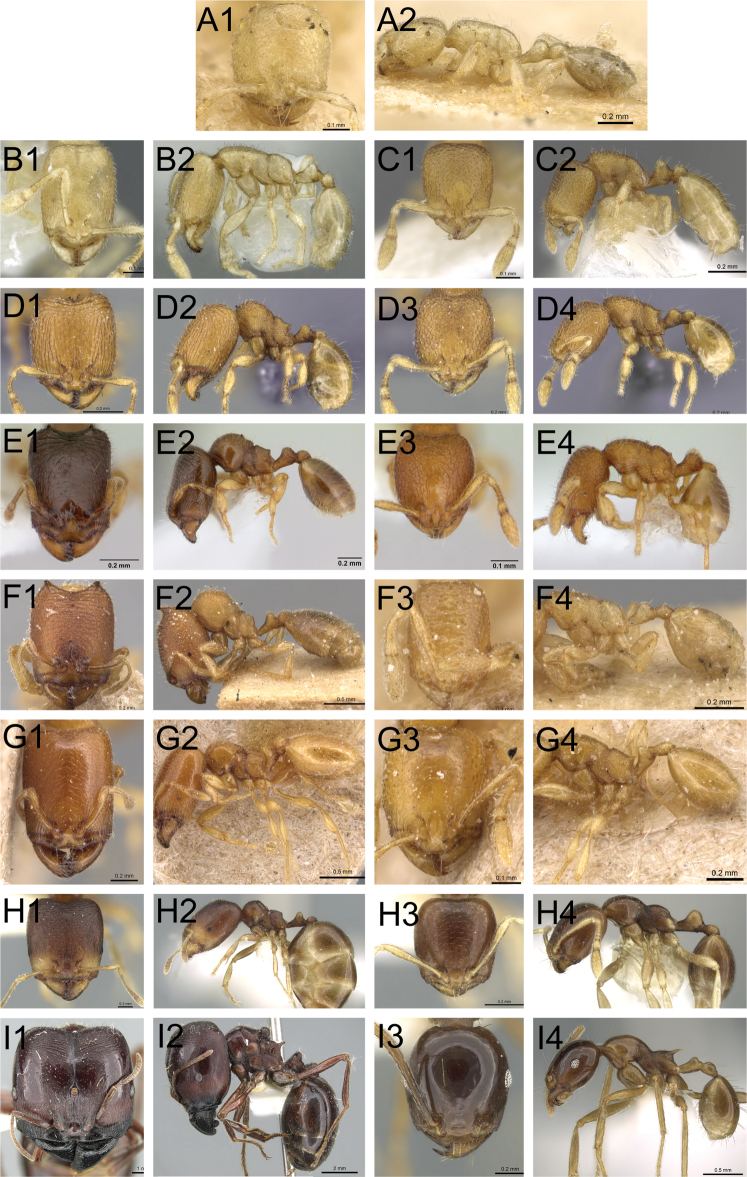
Genera synonymised under *Carebara*, illustrating some of the morphologial diversity expressed across the genus, with each specimen shown in full-face view and in profile. *Carebara*: *Carebara lignata* minor worker: CASENT0902404 (**A1, A2**). *Parvymyrma*: *Carebara sangi* minor worker: CASENT0905888 (**B1, B2**). *Paedalgus*: *Carebara pisinna* minor worker: CASENT0902374 (**C1, C2**). *Carebara*: *Carebara spinata* major worker: CASENT0906099 (**D1, D2**); minor worker: CASENT0906101 (**D3, D4**). *Carebara*: *Carebara intermedia* major worker: CASENT0603534 (**E1, E2**); minor worker: CASENT0603535 (**E3, E4**). *Oligomyrmex (Aneleus)*: *Carebara diabola* major worker: CASENT0904663 (**F1, F2**); minor worker: CASENT0904664 (**F3, F4**). *Pheidologeton (Aneleus)*: *Carebara perpusilla* major worker: CASENT0904661 (**G1, G2**); minor worker: CASENT0904662 (**G3, G4**). *Pheidologeton*: *Carebara pygmaea* major worker: CASENT0906203 (**H1, H2**); minor worker: CASENT0906214 (**H3, H4**). *Pheidologeton*: *Carebara diversa* major worker: CASENT0906200 (**I1, I2**); minor worker: CASENT0906201 (**I3, I4**).

### Indices

CI cephalic index: HW / HL × 100

SI scape index: SL / HW × 100

MDI mandible index: MDL / HW × 100

EI eye index: EL / HW × 100

FI metafemur index: MFL / HW × 100.

PSLI propodeal spine index: PSL / HW × 100

LPpI lateral postpetiole index: PPL / PPH × 100

DPpI dorsal postpetiole index: PPW / PPL × 100

PpWI postpetiole width index: PPW / PTW × 100

PpLI postpetiole length index: PPL / PTL ×100

PpHI postpetiole height index: PPH / PTH × 100

PPI postpetiole index: PPW / PNW × 100

## Results

### Synonymic list of the genus *Carebara*

***Pheidologeton*** Mayr, 1862: 750. Type species: *Oecodoma diversa* Jerdon, 1851: 109, by subsequent designation of Bingham 1903: 160. **syn. n.**

***Oligomyrmex*** Mayr, 1867: 110. Type species: *Oligomyrmex concinnus* Mayr, 1867: 111, by monotypy. [Junior synonym of *Carebara*: Fernández 2004: 194].

***Aeromyrma*** Forel, 1891: 198. Type species: *Aeromyrma nosindambo* Forel, 1891: 199, by monotypy. [Subgenus of *Oligomyrmex*: Emery 1915: 59; revived as genus: Arnold 1916: 256; maintained as genus: Wheeler 1922a: 663, 882; junior synonym of *Oligomyrmex*: Ettershank 1966: 119; junior synonym of *Carebara*: Fernández 2004: 194.]

***Aneleus*** Emery, 1900: 327 (as subgenus of *Pheidologeton*). Type species: *Solenopsis similis* Mayr, 1862: 751, by designation of Wheeler 1911: 158. [Raised to genus: Emery 1914: 41; maintained as genus: Arnold 1916: 254; Forel 1917: 243; Wheeler 1922a: 663; Emery 1924: 213. Senior synonym of *Sporocleptes*: Consani 1951: 169; Arnold 1952: 460. Junior synonym of *Oligomyrmex*: Ettershank 1966: 119; junior synonym of *Carebara*: Fernández 2004: 194.]

***Erebomyrma*** Wheeler, 1903: 138. Type species: *Erebomyrma longii* Wheeler, 1903: 140, by monotypy. [Senior synonym of *Spelaeomyrmex*: Wilson 1962: 63; junior synonym of *Oligomyrmex*: Ettershank 1966: 119; revived as valid genus: Wilson 1986: 61; returned to synonymy of *Oligomyrmex*: Bolton 1994: 106; junior synonym of *Carebara*: Fernández 2004: 194.]

***Phidologeton*** Bingham, 1903: 160. *Phidologeton* as junior synonym of *Pheidologeton*: Wheeler 1922a: 880. syn. n.

***Paedalgus*** Forel, 1911a: 217. Type species: *Paedalgus escherichi* Forel, 1911a: 218. [Junior synonym of *Carebara*: Fernández 2004: 235.]

***Lecanomyrma*** Forel, 1913c: 56 (as subgenus of *Pheidologeton*). Type species: *Pheidologeton (Lecanomyrma) butteli* Forel, 1913c: 56, by monotypy. [Subgenus of *Aneleus*: Emery 1924: 215; junior synonym of *Oligomyrmex*: Ettershank 1966: 123; junior synonym of *Carebara*: Fernández 2004: 235.]

***Spelaeomyrmex*** Wheeler, 1922b: 9. Type species: *Spelaeomyrmex urichi* Wheeler, 1922b: 45, (Fig. 1), by original designation. [Junior synonym of *Erebomyrma*: Wilson 1962: 63; junior synonym of *Carebara*: Fernández 2004: 194.]

***Hendecatella*** Wheeler, 1927: 93 (as subgenus of *Oligomyrmex*). Type species: *Oligomyrmex (Hendecatella) capreolus* Wheeler, 1927: 93, by monotypy. [Junior synonym of *Oligomyrmex*: Ettershank 1966: 119; junior synonym of *Carebara*: Fernández 2004: 194.]

***Amauromyrmex*** Wheeler, 1929a: 1. Type species: *Amauromyrmex speculifrons* Wheeler, 1929a: 1 (junior synonym of *Pheidole silenus* Smith: Ettershank 1966: 119). [*Amauromyrmex* as junior synonym of *Pheidologeton*: Ettershank 1966: 115.] syn. n.

***Solenops*** Karavaiev, 1930: 207 (as subgenus of *Solenopsis*). Type species: *Solenopsis (Solenops) weyeri* Karavaiev, 1930: 207, by monotypy. [Junior synonym of *Oligomyrmex*: Ettershank 1966: 119; junior synonym of *Carebara*: Fernández 2004: 194.]

***Idrisella*** Santschi, 1937c: 372. Type species: *Pheidologeton dentiviris* Forel, 1913b: 192, by original designation. [*Idrisella* as junior synonym of *Pheidologeton*: Ettershank 1966: 115.] **syn. n.**

***Sporocleptes*** Arnold, 1948: 219. Type species: *Sporocleptes nicotianae* Arnold, 1948: 219, by original designation [Junior synonym of *Aneleus*: Consani 1951: 169; Arnold 1952: 460; junior synonym of *Oligomyrmex*: Ettershank 1966: 119; junior synonym of *Carebara*: Fernández 2004: 194.]

***Crateropsis*** Patrizi, 1948: 174 (as subgenus of *Solenopsis*). Type species: *Solenopsis (Crateropsis) elmenteitae* Patrizi, 1948: 174, by monotypy. [Junior synonym of *Oligomyrmex*: Ettershank 1966: 120; junior synonym of *Carebara*: Fernández 2004: 194.]

***Nimbamyrma*** Bernard, 1953: 241. Type species: *Nimbamyrma villiersi* Bernard, 1953: 241. [Junior synonym of *Oligomyrmex*: Ettershank 1966: 120; junior synonym of *Carebara*: Fernández 2004: 235.]

***Afroxyidris*** Belshaw & Bolton, 1994: 631. Type species: *Afroxyidris crigensis* Belshaw & Bolton, 1994: 632, by original designation. [Junior synonym of *Carebara*: Fernández 2004: 235.]

***Neoblepharidatta*** Sheela & Narendran, 1997: 89. Type species: *Neoblepharidatta nayana* Sheela & Narendran, 1997: 89, by original designation. [Junior synonym of *Oligomyrmex*: Bolton 2003: 273; junior synonym of *Carebara*: Fernández 2004: 196.]

***Parvimyrma*** Eguchi & Bui, 2007: 44. Type species: *Parvimyrma sangi* Eguchi & Bui, 2007: 44, by original designation. [Junior synonym of Carebara: Fernández 2010: 235.]

### *Pheidologeton* Mayr – a junior synonym of *Carebara* Forel

Kugler (1986), by studying their sting apparatus, suggested that *Pheidologeton pygmaeus* (*Carebara pygmaea* comb. n.) and *Carebara overbecki*, and *Carebara silena* comb. n. and *Carebara urichi* respectively, must be closely related. Fernández (2010) pointed out the similarity between *Carebara (Nimbamyrma) villiersi* and *Pheidologeton* workers. There are also strong similarities between *Carebara alperti* and *Carebara pygmaea* comb. n., *Carebara rubra* comb. n., and *Carebara transversalis* comb. n. workers. Since all former *Pheidologeton* species are dimorphic or polymorphic it seems very likely that *Carebara alperti* is dimorphic or polymorphic as well. The *Carebara polita* species group here established possesses several morphological features that closely link the former *Pheidologeton* with *Carebara*, the only morphological difference being the prescence of an elongated postpetiole in the *Carebara polita* group versus a rounded or globular postpetiole in *Pheidologeton* workers. We found that the species of the *Carebara polita* group are most similar to the following former *Pheidologeton* species: *Carebara aberrans* comb. n., *Carebara affinis* comb. n., *Carebara diversa* comb. n., *Carebara kunensis* comb. n., *Carebara melanocephala* comb. n., *Carebara nana* comb. n., and *Carebara silena* comb. n.. Our morphological analyses are strongly supported by a recent molecular phylogeny of the subfamily Myrmicinae as part of the Ant Tree of Live project (AToL), which found that *Pheidologeton affinis* (old combination) is nested within *Carebara* (*Carebara alperti*, *Carebara urichi*, and *Carebara vidua*), coupled with the conclusion that *Carebara* is sister to the genus *Diplomorium* (Ward et al. 2014).

These independently generated results finally led us to the current synonymization of *Pheidologeton* under *Carebara*, as proposed in the present publication. As a result of the synonymization, *Carebara* becomes a morphologically and ecologically highly diverse genus, with a variety of monomorphic, dimorphic and polymorphic species. However, it is possible that the majority of *Carebara* species have at least dimorphic or even potentially polymorphic workers, since recent collections show that worker dimorphism is common in species that were previously known as monomorphic, and polymorphism is common in species that were previously known as dimorphic (Azorsa and Fisher, in review; GF and FA, unpublished). We speculate that species in the *polita* group and in other *Carebara* groups that are currently only known from minor workers may produce major workers infrequently or only on specific environmental or genetic cues that are yet unstudied. Despite a large number of collections from a large distribution range across sub-Saharan Africa, the major workers of *Carebara polita* remained undescribed for 100 years and the earliest collection with major workers is from 2001 (by R.R. Snelling) and only from Kakamega Forest in Western Kenya. Alternatively, the need to maintain major workers may have subsided during the evolution of some species or even whole species groups, resulting in a complete loss or reduction of worker polymorphism.

There are other examples of species within *Carebara* that are morphological links between other previously separated genera (Fernández 2004; 2010), such as between *Carebara spinata* and *Carebara intermedia* and the former *Paedalgus*, the only difference between them being armed versus an unarmed propodeum. An unarmed propodeum, however, is not synapomorphic for *Paedalgus*, because it is present in several other species of *Carebara* and in the former *Oligomyrmex*. Similarly, the only known species of the former genus *Parvimyrma* (*Carebara sangi*), described by Eguchi and Bui (2007), and included in *Carebara* by Fernández (2010), is closely related to workers of the dimorphic *Carebara diabola*, as well as to *Carebara carinata*. The elongated head and the shapes of the propodeum and petiole are almost the same in workers of all three species. It is difficult to see whether the type specimens of *Carebara diabola* have a central clypeal hair, but regardless, the state of this character (one hair in *Carebara sangi* versus two central clypeal hairs in other *Carebara*) is not sufficient to treat *Parvimyrma* as a separate genus. A single central clypeal hair is also present in *Carebara carinata*.

Due to the considerable amount of morphological diversity across the genus *Carebara*, its workers can be distinguished from those of other myrmicine genera with a two-segmented antennal club only by a combination of the following characters.

### Diagnostic characters for *Carebara* workers (revised to include former *Pheidologeton* species)

1. Antenna with eight to eleven segments and a two-segmented club.

2. Clypeus of minor workers usually with four distinct setae (some species with a central isolated seta).

3. Mandibles of workers triangular or subtriangular and usually with four to seven teeth or denticles present (3 teeth in *Carebara crigensis* (Belshaw and Bolton 1994)), the apical and preapical tooth often larger than the following ones.

4. Palp formula 2,2 or 1,2.

5. Frontal lobes separated by median part of clypeus.

6. Eyes either absent or reduced to one or few ommatidia and situated anterior of cephalic midlength, or larger, not reduced, and situated at or posterior of cephalic midlength.

7. Antennal scrobes absent, weakly developed only in species with workers with phragmotic head.

8. Frontal carinae varying from absent to weakly developed and short to very rarely reaching beyond cephalic midlength, but never extending towards posterior head margin.

9. Promesonotal dorsum in profile convex to weakly convex, very rarely near-linear.

10. Propodeum often with a pair of spines, short teeth or angulate posterior corners, in which cases often with a lamella reaching down towards propodeal lobes, but sometimes propodeum posteriorly completely unarmed and rounded.

11. Petiole with a distinct peduncle and well-differentiated node.

12. Different worker subcastes in dimorphic and polymorphic species, sometimes with enormous size variation (e.g. in *Carebara polita* group and in many former *Pheidologeton*).

13. Major workers, where present and especially when large, often with at least a few small remnants of queen flight sclerites present, and some *polita* group and former *Pheidologeton* major workers with all flight scerites recognizable.

### Comments on diagnosis of *Carebara* worker caste

*Carebara* workers may be monomorphic, dimorphic, or continuously polymorphic. When the latter is the case, there are often several subcastes intermediate between minor and large major workers. Intermediate workers of some marauder ant species in the former genus *Pheidologeton* differ gradually in size and general morphology. In other species with polymorphic workers (e.g. in several former *Oligomyrmex*) the morphological differences are less gradual, with little variation in the minor workers, but with one to several different major worker phenotypes that differ in size and morphology.

Some species, like *Carebara elmenteitae* from Kenya, *Carebara nayana* from India and *Carebara butteli* from Sri Lanka, however, have an additional major worker subcaste with phragmotic heads, which represent a special defense line against predators and are often described as living plugs for nest entrances. Interestingly *Carebara butteli* (Forel 1913c) was originally described as *Pheidologeton butteli*.

### Species groups in *Carebara*

The establishment of the *Carebara polita* species group highlights the need to revise all *Carebara* species to better delimit species group boundaries in the genus and to rearrange the groups suggested by Fernández (2004, 2010) and later adapted to include the Indian *Carebara* fauna treated by Bharti and Kumar (2013). The establishment of new species groups will likely be necessary with ongoing taxonomic research and will help unravel the evolutionary history of this genus. Especially the Afrotopical region holds a large number of unrevised and new species, many of which are located in the BMNH collection in London.

The former *Pheidologeton* species could in future revisions be split into two species groups, one that shares characters with *Carebara aberrans* comb. n. and *Carebara affinis* comb. n., where the minor and large major workers are connected through a continuous series of intermediate subcastes, and another group that shares characters with *Carebara pygmaea* comb. n. and *Carebara alperti*, that are dimorphic and morphologically similar to some species of the former genus *Oligomyrmex*. In this publication we are establishing and focusing on the *polita* species group, which is mostly characterized by their minor workers’ morphology, i.e. head and mesosoma shape, medially smooth and shiny frons, present propodeal spines, and, most importantly, an elongate postpetiole in minor workers. The diagnosis for minor and major workers (where known) of the *polita* group is summarized below.

### Diagnostic characters of the *polita* species group

1. Antennae with usually eleven, but one species with nine segments; scapes, when laid back, never reaching or surpassing posterior head margin.

2. Eyes of minor workers small, in most specimens consisting of only one ommatidium, in major workers absent to multi-facetted, in all subcastes situated anterior to cephalic midlength and relatively close to anterior head margin.

3. Madibles with four to six teeth, number in minor workers often one less than in major workers.

4. Posterior margin of head concave to weakly concave medially, or nearly straight.

5. Frons in minor workers medially smooth and shiny.

6. Minor workers with short to comparatively long propodeal spines present, in major workers sometimes reduced to a blunted angle (*Carebara urichi*).

7. Minor and/or major workers often with petiolar ventral process present as anteriorly directed small tooth, which is sometimes reduced or inconspicuous.

8. Petiole node in minor workers usually subangulate to rounded in profile, dorsally smooth and shiny, in major workers very well-developed and high, the dorsum rounded to angulate-subangulate and anterodorsally compressed.

9. Minor worker postpetiole relatively elongate, distincly longer than high (LPpI 131–173) and lower than petiole, major worker postpetiole compact, dorsally rounded, and in profile about as high as long (LPpI 83–107).

### Comments on diagnosis of *polita* group worker caste

Range in head shape and sculpture, the number of distinctly expressed mesosomal sclerites in major workers, and the propodeum varying from having spines or not in this group is relatively large and comparable to the range found in former *Pheidologeton* species. Interestingly the major worker caste has shown itself completely elusive in two of the *polita* group species. Despite a multitude of collections from several localities *Carebara brevipilosa* and *Carebara villiersi* are known only from the minor workers so far. But *Carebara polita* has been collected without any major workers for almost a century, and *Carebara nicotianae* for more than 50 years. The minor workers of *Carebara polita* were described by F. Santschi in 1914 and majors have not been collected until 2001 by the late R. R. Snelling, but remained unidentified at first. If not for another collection of major workers together with queens and minor workers in 2008 by the first author, the fact that these two different worker subcastes belong to the same species, and one important link between *Carebara* and the former *Pheidologeton*, might have remained unnoticed.

### Biogeographic notes on the *polita* species group

Six species occur in the Afrotropical region, two of them are widely distributed throughout sub-Saharan Africa: *Carebara perpusilla* in Kenya, Rwanda, South Africa, Tanzania, Uganda, Zambia, and Zimbabwe and *Carebara silvestrii* in Cameroon, Central African Republic, Equatorial Guinea, Gabon, Ghana, Ivory Coast, Kenya, Uganda, and Zimbabwe. Two species are widely distributed from Western to Eastern Africa: *Carebara polita* in Cameroon, Central African Republic, Gabon, Kenya, Tanzania, and Uganda and *Carebara madibai* sp. n. is known from the Central African Republic, Dem. Rep. Congo, Gabon, and Uganda. *Carebara villiersi* occurs throughout Western Africa and was found in Cameroon, Central African Republic, Gabon, Ghana, Guinea, and Ivory Coast. *Carebara nicotianae* seems restricted to Zambia and Zimbabwe in Southern Africa. Two of the species included in this group are present and widespread in the Neotropical Region: *Carebara brevipilosa* is reported from Brazil, Colombia, Costa Rica, and Panama, while *Carebara urichi* was found only in Belize, Brazil, Colombia, Costa Rica, Mexico, Panama, Peru, Suriname, and Trinidad.

### Synopsis of the *polita* species group

*Carebara brevipilosa* Fernández, 2004

*Carebara madibai* Fischer & Azorsa, sp. n.

*Carebara perpusilla* (Emery, 1895)

= *Oligomyrmex perpusillus arnoldianus* Ettershank, 1966 syn. n. [Replacement name for *Pheidologeton perpusillum* subsp. *arnoldi* Forel: Ettershank 1966: 123. Junior secondary homonym of *Oligomyrmex arnoldi* Forel 1913a: 123.]

= *Pheidologeton (Aneleus) perpusillus spinosus* Forel, 1907, syn. n.

= *Aneleus perpusillus concedens* Santschi, 1914a, syn. n.

*Carebara polita* (Santschi, 1914a)

*Carebara nicotianae* Arnold, 1948, comb. n. et stat. rev.

*Carebara silvestrii* (Santschi, 1914b), comb. n.

= *Aneleus (Aneleus) punctatus* Karavaiev, 1931, syn. n.

*Carebara urichi* (Wheeler, 1922b)

= *Erebomyrma nevermanni* Mann, 1926

= *Erebomyrma morai* Menozzi, 1931

= *Erebomyrma eidmanni* Eidmann, 1936

*Carebara villiersi* (Bernard, 1953)

### New combinations for all former species of *Pheidologeton* synonymised under *Carebara*

Where necessary, the endings of specific epithets were changed in order to agree with the gender of the genus *Carebara*, e.g. as in *Carebara diversa* (Jerdon, 1851) comb. n. – previously *Pheidologeton diversus*. To avoid the creation of secondary junior homonyms, original species names of three former *Pheidologeton* and two species already in *Carebara* are here replaced with new names.

*Carebara aberrans* (Santschi, 1937b), comb. n.

*Carebara affinis* (Jerdon, 1851), comb. n.

*Carebara affinis javana* (Emery, 1893a), comb. n.

*Carebara affinis minor* (Emery, 1900), comb. n.

*Carebara affinis spinosior* (Forel, 1911b), comb. n.

*Carebara affinis sumatrensis* (Forel, 1913c), comb. n.

*Carebara ceylonensis* (Forel, 1911a), comb. n.

*Carebara dentiviris* (Forel, 1913b), comb. n.

*Carebara diversa* (Jerdon, 1851), comb. n.

*Carebara diversa draco* (Santschi, 1920), comb. n.

*Carebara diversa ficta* (Forel, 1911b), comb. n.

*Carebara diversa laotina* (Santschi, 1920), comb. n.

*Carebara diversa macgregori* (Wheeler, 1929b), comb. n.

*Carebara diversa philippina* (Wheeler, 1929b), comb. n.

*Carebara diversa taprobanae* comb. n. (Smith, 1858). [Senior secondary homonym of *Carebara taprobanae* Forel, 1911a, replacement name *Carebara sinhala* nom. n.]

*Carebara diversa standfussi* (Forel, 1911b), comb. n.

*Carebara diversa tenuirugosa* (Wheeler, 1929b), comb. n.

*Carebara diversa williamsi* (Wheeler, 1929b), comb. n.

*Carebara gustavmayri* nom. n. (Replacement name for *Pheidologeton mayri* Santschi, 1928). [Junior] secondary homonym of *Carebara mayri* (Forel, 1901).]

*Carebara hammoniae* (Stitz, 1923), comb. n.

*Carebara hostilis* (Smith, 1858), comb. n.

*Carebara kunensis* (Ettershank, 1966), comb. n.

*Carebara latinoda* (Zhou & Zheng, 1997), comb. n.

*Carebara luzonensis* nom. n. (Replacement name for *Pheidologeton silvestrii* Wheeler, 1929b). [Junior secondary homonym of *Carebara silvestrii* (Santschi, 1914b).]

*Carebara maccus* (Wheeler, 1929b), comb. n.

*Carebara melanocephala* (Donisthorpe, 1948), comb. n.

*Carebara melasolena* (Zhou & Zheng, 1997), comb. n.

*Carebara nana* (Roger, 1863), comb. n. [Senior secondary homonym of *Carebara nana* (Santschi, 1919), replacement name *Carebara pumilia* nom. n.]

*Carebara nanningensis* (Li & Tang, 1986), comb. n.

*Carebara obscura* (Viehmeyer, 1914), comb. n.

*Carebara petulens* (Santschi, 1920), comb. n.

*Carebara pullata* (Santschi, 1920), comb. n.

*Carebara pungens* (Smith, 1861), comb. n.

*Carebara pygmaea* (Emery, 1887), comb. n. (Syntypes: 1 major & 4 minor workers. Emery 1887: 465. INDONESIA: Ternate Acqui Conora xi. 1874 (*Beccari*) [MHNG] [examined]).

= *Pheidologeton pygmaeus albipes* Emery, 1893b, comb. n., syn. n. (Holotype worker. Emery 1893b: 266. PHILIPPINES: Antipolo [MSNG] [examined]).

= *Carebara simularensis* Forel, 1915, syn. n. (Syntypes: 3 major & 3 minor workers, 1 queen. Forel 1915: 27. INDONESIA: Sinabang, Sumatra 47, No. 13. i.1913 (*E. Jacobson*) [MHNG] [examined]).

= *Pheidologeton (Aneleus) pygmaeus bugnioni* Forel, 1915, comb. n., syn. n. (Syntypes: 5 major & 5 minor workers. Forel 1915: 28. SRI LANKA: Peradeniya (*Bugneon*) [MHNG] [examined]).

*Carebara rubra* (Smith, 1860b), comb. n.

† *Carebara rugiceps* (Heer, 1849), comb. n.

† *Carebara schossnicensis* (Assmann, 1870), comb. n.

*Carebara rugoflabella* nom. n. (Replacement name for *Pheidologeton rugosus* Karavaiev, 1935). [Junior secondary homonym of *Carebara ampla rugosa* (Santschi, 1928).]

*Carebara silena* (Smith, 1858), comb. n.

*Carebara solitaria* (Stitz, 1910), comb. n.

*Carebara transversalis* (Smith, 1860a), comb. n.

*Carebara trechideros* (Zhou & Zheng, 1997), comb. n.

*Carebara varia* (Santschi, 1920), comb. n.

*Carebara vespilla* (Wheeler, 1921), comb. n.

*Carebara volsellata* (Santschi, 1937a), comb. n.

*Carebara yanoi* (Forel, 1912), comb. n.

*Carebara zengchengensis* (Zhou, Zhao & Jia, 2006), comb. n.

### Identification key for the species of the *polita* species group

[Combined key for major and minor workers, but note that majors of *Carebara brevipilosa* and *Carebara villiersi* are unknown.]

**Table:** 

1a	Antennae with 11 segments. **Major workers** (where known): Head of major in full-face view either with subparallel sides or posterior wider than anterior (Fig. 3A). **Minor workers:** Either frontal carinae extending to about cephalic midlength or less (Fig. 3B), or if longer, then dorsum of promesonotum at least partly with sculpture	2
1b	Antennae with 9 segments. **Major workers:** Head anterior significantly wider than posterior (Fig. 3C). **Minor workers:** Frontal carinae extending to about ¾ of the length of the head (Fig. 3D) and dorsum of promesonotum smooth and shiny. (Central African Republic, Dem. Rep. Congo, Gabon, Uganda)	*Carebara madibai*

**Figure 3.**
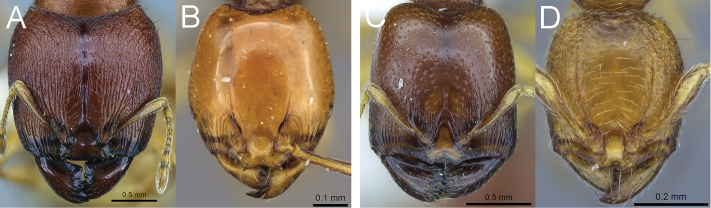
Full-face view of *Carebara polita* major worker (**A**) and minor worker (**B**); full-facev view of *Carebara madibai* major worker (**C**) and minor worker (**D**).

**Table:** 

2a	**Major workers:** Head posterior wider than anterior, in full-face view weakly heart-shaped or with angulate posterolateral corners (Fig. 4A). **Minor workers:** Head entirely smooth and shiny and oval, with convex to strongly convex sides and rounded posterior corners (Fig. 4B)	3
2b	**Major workers:** Head posterior about as wide as anterior, with subparallel sides and rounded posterolateral corners (Fig. 4C). **Minor workers:** Head either not entirely smooth and shiny, or subrectangular with weakly convex sides, or both (Fig. 4D)	5

**Figure 4.**
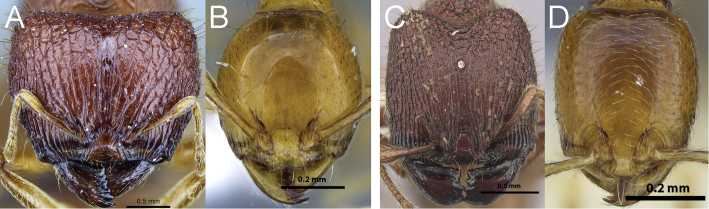
Full-face view of *Carebara nicotianae* major worker (**A**) and *Carebara villiersi* minor worker (**B**); full-face view of *Carebara urichi* major worker (**C**) and *Carebara perpusilla* minor worker (**D**).

**Table:** 

3a	**Major workers:** Head and body very large and massive (HW 1.20–2.09, PNH 0.83–1.48), head posterior wider than anterior with convex sides and widely concave to shallowly V-shaped posterior emargination (Fig. 5A, B). **Minor workers:** Head oval with strongly convex sides, posterior head margin deeply concave in full-face view, frontal carinae moderately long, reaching to about cephalic midlength or slightly beyond (Fig. 5C)	4
3b	**Major workers:** unknown. **Minor workers:** Head shape rounded with strongly convex sides, with posterior head margin straight to faintly convex medially, frontal carinae very short to inconspicuous (Fig. 5D), spines very long, in dorsal view distinctly longer than space between them. (Cameroon, Central African Republic, Gabon, Ghana, Guinea, Ivory Coast)	*Carebara villiersi*

**Figure 5.**
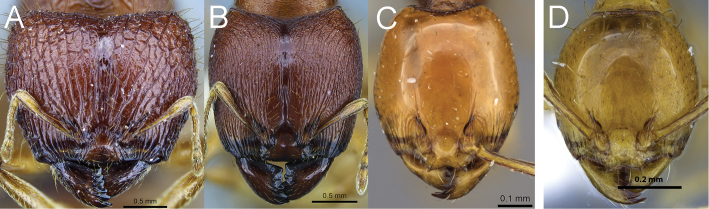
Full-face view of *Carebara nicotianae* major worker (**A**), *Carebara polita* major worker (**B**), and *Carebara polita* minor worker (**C**); full-face view of *Carebara villiersi* minor worker (**D**).

**Table:** 

4a	**Major workers:** Head posterior with small protuberances or horns at posterolateral lobes, scutum in profile dorsally straight or only faintly convex (Fig. 6A), petiolar anteroventral process shallowly developed, pubescence and standing hairs on gaster rather rare. **Minor workers:** Mesonotum and propodeum in profile mostly smooth and shiny (Fig. 6B). (Cameroon, Central African Republic, Gabon, Kenya, Tanzania, Uganda)	*Carebara polita*
4b	**Major workers:** Head without horns at posterolateral lobes, scutum in profile dorsally strongly convex (Fig. 6C), petiolar anteroventral process well-defined, finger-like, pointing anteriorly, pubescence and standing hairs on gaster very abundant. **Minor workers:** Mesonotum and propodeum in profile areolate (Fig. 6D). (Zambia, Zimbabwe)	*Carebara nicotianae*

**Figure 6.**
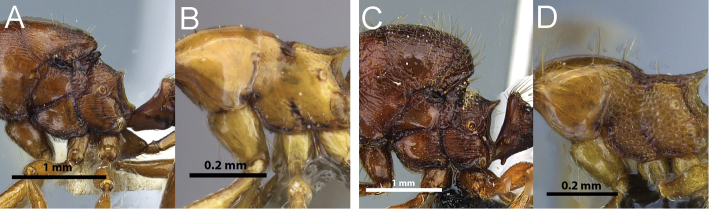
Profile of *Carebara polita* major worker mesosoma and petiole (**A**), profile of *Carebara polita* minor worker mesosoma (**B**); profile of *Carebara nicotianae* major worker mesosoma and petiole (**C**), profile of *Carebara nicotianae* minor worker mesosoma (**D**).

**Table:** 

5a	**Major workers:** Head distinctly longer than wide in full-face view (CI 71–87) (Fig. 7A). Posterior head margin with transverse carina. **Minor workers:** Anteroventral face of petiole straight or weakly concave and subpetiolar process absent (Fig. 7B). (Afrotropics)	6
5b	**Major workers** (*Carebara urichi* only): Head about as long as wide to slightly longer (CI 92–99) (Fig. 7C). Posterior head margin lacking transverse carina. **Minor workers:** Subpetiolar process present (Fig. 7D). (Neotropics)	7

**Figure 7.**
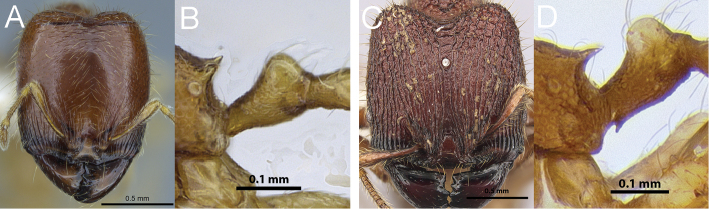
Full-face view of *Carebara silvestrii* largest major worker (**A**), profile of *Carebara perpusilla* minor worker petiole (**B**); full-face view of *Carebara urichi* major worker (**C**), profile of *Carebara urichi* minor worker petiole (**D**).

**Table:** 

6a	Smaller species (HW major worker: 0.52–0.70). **Major workers:** Lateropronotum and anepisternum smooth and shiny, katepisternum and lateropropodeum weakly to superficially areolate (Fig. 8A). Postpetiole relatively wide, compared to pronotal width (PPI 60–66) and postpetiole length (DPpI 100–128). **Minor workers:** Cephalic dorsum and pronotum smooth and shiny, head shape subrectangular with weakly convex sides (Fig. 8B). (Kenya, Rwanda, South Africa, Tanzania, Uganda, Zambia, Zimbabwe)	*Carebara perpusilla*
6b	Slightly larger species (HW major worker: 0.64–0.99). **Major workers:** Lateropronotum, anepisternum, katepisternum, and lateropropodeum usually densely areolate (Fig. 8C). Postpetiole relatively narrower, compared to pronotal width (PPI 45–52) and postpetiole length (DPpI 132–167). **Minor workers:** Frons often smooth and shiny, remainder of cephalic dorsum usually coarsely rugoreticulate, pronotum never completely smooth, usually with some areolate or rugose sculpture present, head shape suboval with strongly convex sides (Fig. 8D). (Cameroon, Central African Republic, Equatorial Guinea, Gabon, Ghana, Ivory Coast, Kenya, Uganda, Zimbabwe)	*Carebara silvestrii*

**Figure 8.**
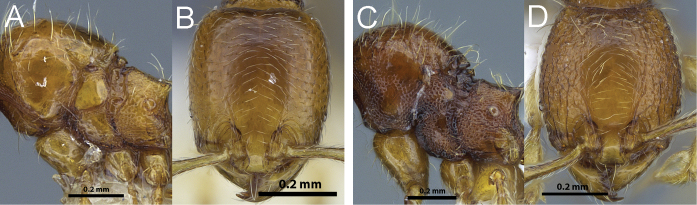
Profile of *Carebara perpusilla* major worker mesosoma (**A**), full-face view of *Carebara perpusilla* minor worker (**B**); profile of *Carebara silvestrii* major worker mesosoma (**C**), full-face view of *Carebara silvestrii* minor worker (**D**).

**Table:** 

7a	**Major workers:** unknown. **Minor workers:** Sculpture on dorsal promesonotum weakly to superficially reticulate with or without very few short rugulae (Fig. 9A), metatibiae with short, appressed to decumbent pilosity (Fig. 9B). (Brazil, Colombia, Costa Rica, Panama)	*Carebara brevipilosa*
7b	**Major workers:** Head with strongly raised rugae anteriorly and rugoreticulation posteriorly (C), metatibiae with subdecumbent pilosity and with long suberect hairs along outer edge. **Minor workers:** Sculpture on dorsal promesonotum irregularly longitudinally rugose to rugoreticulate with few irregular longitudinal rugae (Fig. 9D), metatibiae with subdecumbent pilosity and additionally with long suberect hairs (Fig. 9E). (Belize, Brazil, Colombia, Costa Rica, Mexico, Panama, Peru, Surinam, Trinidad)	*Carebara urichi*

**Figure 9.**
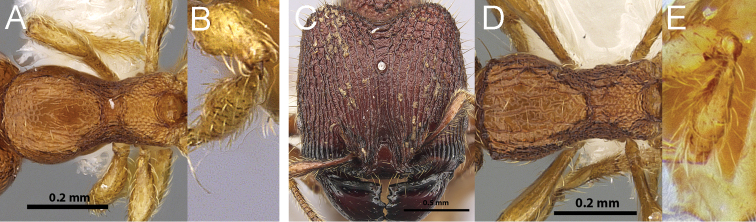
Dorsal view of *Carebara brevipilosa* minor worker mesosoma (**A**), profile of minor worker metatibia (**B**); full-face view of *Carebara urichi* major worker (**C**), dorsal view of *Carebara urichi* minor worker mesosoma (**D**), profile of minor worker metatibia (**E**).

## Species accounts

### Carebara brevipilosa

Taxon classificationAnimaliaHymenopteraFormicidae

Fernández

Figure 10

Carebara brevipilosa Fernández, 2004: 210. Holotype worker: COLOMBIA: Caquetá, San José de la Fragua, La Esmeralda, Yuruyaco River, 1500m, 7-10.ix.2002 (*E.L. González*) (IAvH), [examined].

#### Diagnosis.

Antennae with 11 segments. **Major workers:** unknown. **Minor worker:** Head almost as wide as long, with longitudinal rugulae and reticulations, except for smooth and shiny frons, petiole anteroventrally with small anteriorly directed tooth, dorsal promesonotum weakly to superficially rugoreticulate, and sometimes with a few weak to superficial rugulae present and metatibiae with short appressed to decumbent pilosity.

**Figure 10.**
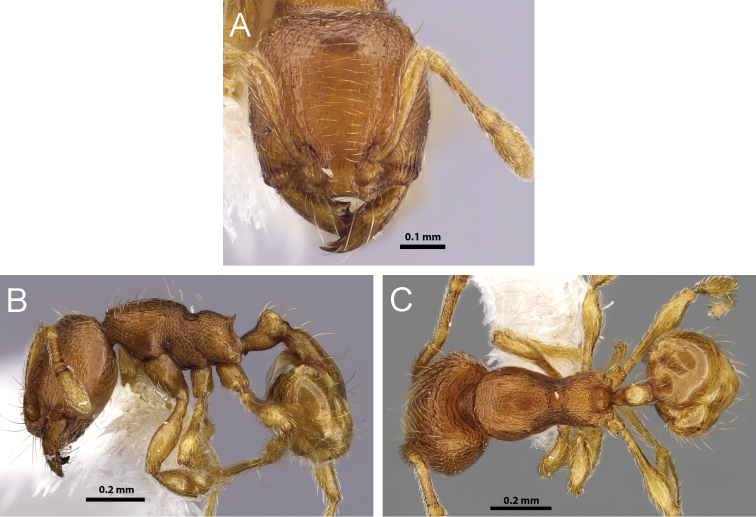
*Carebara brevipilosa*. Minor worker, CASENT0609948: **A** head in full-face view **B** body in profile **C** body in dorsal view.

#### Description of minor workers.

Measurements (n=5): HW 0.31–0.32 (0.31), HL 0.32–0.33 (0.32), SL 0.20–0.21 (0.21), MDL 0.20-0.21 (0.20), EL 0.01–0.02 (0.01), WL 0.35–0.37 (0.35), PNH 0.14–0.16 (0.15), PNW 0.19–0.20 (0.19), MNH 0.20, PDH 0.14, PTL 0.13–0.14 (0.14), PPL 0.07–0.08 (0.08), PTH 0.09–0.11 (0.10), PPH 0.05–0.06 (0.05), PTW 0.05, PPW 0.08–0.09 (0.08), PSL 0.04–0.05 (0.04), MFL 0.23–0.25 (0.24), MTL 0.17–0.19 (0.18), CI 95–98 (97), SI 65–68 (67), MDI 65–67 (66), EI 4–5 (5), FI 74–80 (77), PSLI 12–15 (14), LPpI 136–143 (139), DPpI 100–116 (108), PpWI 157–190 (170), PpLI 53–58 (55), PpHI 50–67 (56).

Head in full-face view almost as wide as long (CI 95–98), narrowed anteriorly and posteriorly, posterior margin straight or feebly concave medially, posterolateral corners bluntly angulate, sides convex. Mandibles with four teeth, apical and preapical tooth larger than following teeth. Clypeus anteriorly concave, bicarinate, subangulate toward sides. Antennae with eleven segments, scapes failing to reach posterior margin of head by about the length of the preapical funicular segment. Eyes reduced to a single ommatidium. Frontal carinae present, usually feebly developed but long, in some specimens almost reaching posterior head margin.

In profile, promesonotum convex or weakly convex, metanotal groove broadly concave and deeply impressed. Dorsum of propodeum almost straight in profile and sloping posteriorly, anterodorsal corner convex, propodeal spines relatively short, triangular and upwardly directed, declivity of propodeum concave. Propodeal spiracle near posterior border of propodeum.

Petiole moderately long, peduncle in profile about as long as petiole node, ventral face convex, anteroventral corner with a small triangular tooth which, in some specimens, can be reduced and inconspicuous, petiole node broadly wedge-shaped and rounded dorsally. Postpetiole in profile weakly convex dorsally, almost straight ventrally, about 1.4 times longer than high (LPpI 136–143) and much lower than petiole (PpHI 50–67). In dorsal view, petiole node almost as wide as long, and roundly convex, postpetiole on average 1.7 times wider than petiole (PpWI 157–190), with convex sides and posteriorly wider than anteriorly.

Head with some irregular, longitudinal striations and rugosities, except for smooth and shiny frons. Mandibles and median portion of clypeus smooth and shiny with scattered punctures, face with scattered punctuations. Mesosoma, petiole and postpetiole areolate-rugose, except for smooth spot on anteroventral pronotum, and smooth and shiny petiole and postpetiole dorsum. Gaster smooth and shiny.

Head and body with relatively few long, suberect standing hairs and with decumbent to subdecumbent short pilosity. Scapes with abundant decumbent pilosity. Four longer hairs on clypeal margin extending close to the anterior border of mandibles. Color dark orange, legs and antennae lighter colored orange.

#### Distribution and biology.

*Carebara brevipilosa* is relatively widespread in the Neotropical Region, found in Brazil, Colombia, Costa Rica, and Panama. This species was collected mainly in the rainforest and at elevations ranging from 50–1050 m. Individuals and nest series were collected from the leaf-litter and soil, using Winkler extractors and pitfall traps.

#### Comments.

*Carebara brevipilosa* can be confused with *Carebara urichi*, but is easily distinguished by the lack of long suberect hairs at the outer edge of the metatibiae and the sculpture on the dorsal promesonotum, which is typically irregularly longitudinally rugose to rugoreticulate with few irregular longitudinal rugae in *Carebara urichi* and weakly to superficially reticulate without or with very few short rugulae in *Carebara brevipilosa*. Also, *Carebara brevipilosa* (HW minor workers 0.41–44, WL 0.48–0.57) seems to be distinctly smaller than *Carebara urichi* (HW minor workers 0.31–0.32, WL 0.35–3.37). These are the only two species in the *polita* group recorded from the Neotropical Region. Major workers of *Carebara brevipilosa* have not been collected or identified yet.

#### Material examined.

**COLOMBIA:** Caquetá, San José de la Fragua, La Esmeralda, Yuruyaco River, 1500 m, 7–10.ix2002 (*E.L. González*); Magdalena, 4km N San Pedro, 10.95, -74.05, 550m, 14.viii.1985 (*P.S. Ward*); **PANAMA:** Darieni Cana, 7.716667, -77.2, 800m, 23.viii.1987 (*D.M. Olson*).

### Carebara madibai

Taxon classificationAnimaliaHymenopteraFormicidae

Fischer & Azorsa sp. n.

http://zoobank.org/83A0FC28-0D89-4FC8-8918-A5B7CD64A02E

Figure 11

#### Holotype.

(major worker) CENTRAL AFRICAN REPUBLIC: Parc National Dzanga-Ndoki, Mabéa Bai, 21.4 km 53° NE Bayanga, 3.03333, 16.41, 510m, rainforest, 10–17.v.2001 (*B.L. Fisher*), Collection code BLF4032 (CASC: CASENT0415384).

#### Paratypes.

(8 major workers and 32 minor workers) 8 major workers, same data as holotype: CASC: CASENT0415383, CASENT0415382, CASENT0709000, CASENT0709001, CASENT0709002, CASENT0709003, CASENT0709004, CASENT0709005. 9 minor workers, same data as holotype: CASC: CASENT0415374, CASENT0415375, CASENT0415381. 23 minor workers with same data as holotype but with different collection number, BLF4000. 2 workers: BMNH: CASENT0405752, CASENT0413423. 2 workers: MCZ: CASENT0413424, CASENT0405761. 19 workers: CASC: CASENT0405759, CASENT0402669, CASENT0405760, CASENT0405750, CASENT0405763, CASENT0405751, CASENT0402683, CASENT0405754, CASENT0402671, CASENT0402672, CASENT0402673, CASENT0405765, CASENT0413325, CASENT0405762, CASENT0405764, CASENT0402674, CASENT0405753, CASENT040681, CASENT0413329.

#### Diagnosis.

Antennae with nine segments. **Major worker:** Head almost subrectangular, longer than wide and anteriorly wider than posteriorly, face smooth and shiny and with comparatively large and scattered foveae in frontal view. Head and body with very short, mostly appressed pilosity, lacking long, standing hairs. **Minor worker:** Posterior margin of head nearly straight, frontal carinae reaching towards posterior quarter of head, dorsum of promesonotum smooth and shiny, propodeal dorsum areolate rugose.

**Figure 11.**
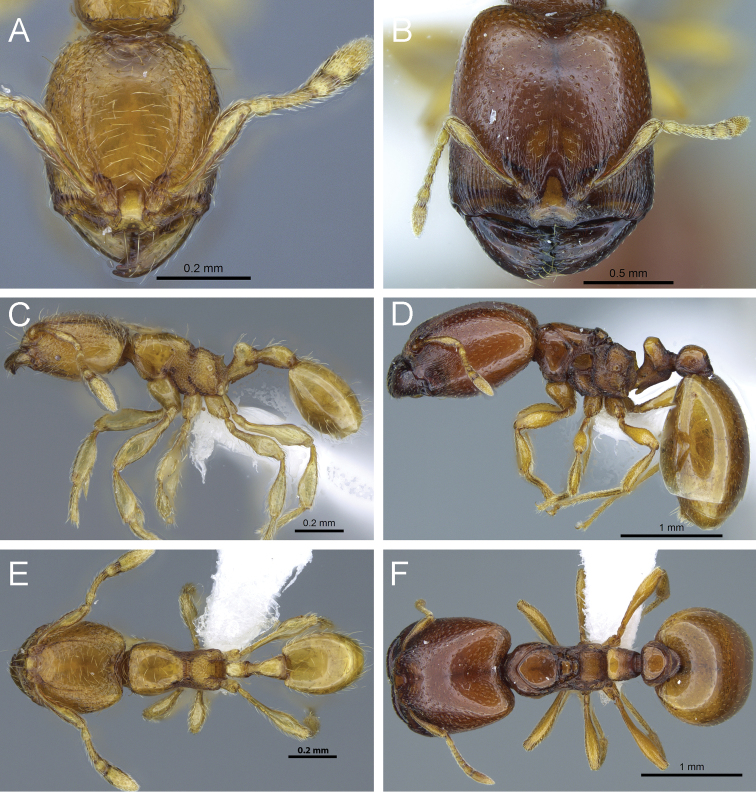
*Carebara madibai*. Paratype minor worker, CASENT0405752: **A** head in full-face view **C** body in profile **E** body in dorsal view. Holotype major worker, CASENT0415384: **B** head in full-face view **D** body in profile **F** body in dorsal view.

#### Description of major workers.

Measurements (n=4): HW 1.09–1.13 (1.12), HL 1.19–1.28 (1.23), SL 0.52–0.54 (0.53), MDL 0.60–0.65 (0.63), EL 0.0, WL 1.02–1.07 (1.04), PNH 0.53–0.56 (0.54), PNW 0.55–0.56 (0.56), MNH 0.72–0.75 (0.74), PDH 0.49–0.51 (0.50), PTL 0.51–0.56 (0.54), PPL 0.32–0.33 (0.32), PTH 0.36–0.44 (0.41), PPH 0.31–0.33 (0.32), PTW 0.33–0.36 (0.34), PPW 0.44–0.50 (0.46), PSL 0.15–0.17 (0.16), MFL 0.69–0.74 (0.72), MTL 0.60–0.63 (0.62), CI 88–92 (91), SI 47–48 (47), MDI 55–58 (56), EI 0, FI 63–65 (64), PSLI 13–15 (15), LPpI 100–102 (101), DPpI 138–150 (143), PpWI 130–141 (135), PpLI 58–62 (60), PpHI 73–88 (78).

Head longer than wide (CI 88–92), in full-face view nearly subrectangular, slightly narrowed posteriorly, posterior margin shallowly concave medially, corners rounded, sides of head straight to weakly convex. Mandibles with five teeth. Frontal carinae absent or inconspicuous. Anterior margin of clypeus weakly concave, sides subangulate. Antennae nine-segmented, scapes short, not surpassing cephalic midlength (SI 47–48). Eyes absent.

In profile, promesonotum nearly straight or weakly convex, slightly higher than propodeum. Scutum well-developed, scutellum present, but smaller than scutum. Promesonotal suture present or absent on dorsum, metanotum present as small and reduced sclerite. Dorsal face of propodeum in profile straight, or slightly concave, declining posteriorly, propodeum with anterodorsal corner weakly rounded, posterior corners with a pair of stout and subtriangular teeth, declivity of propodeum vertical, slightly concave in the middle, lamella well–developed and extending from the propodeal teeth to the propodeal lobe, forming a subangulate triangular shape. Propodeal spiracle rounded, situated close to center of lateral propodeum.

Petiole with moderately long peduncle, ventral face concave and posterior margin convex, subpetiolar process relatively large, forward-directed. In profile, petiole node with anterior margin deeply concave in middle, posterior margin vertical, nearly straight to slightly concave, and dorsal face roundly convex. Petiole node high, and well developed, antero-posteriorly compressed. Postpetiole roundly convex, and lower than petiole. In dorsal view, postpetiole almost as wide as petiole (PPW 0.44–0.50, PTW 0.33–0.36), petiole node wider than long, and flattened anteroposteriorly, anterior face of petiole nearly straight, posterior face roundly convex, sides rounded, postpetiole wider than long, anterior face nearly straight and posterior face roundly convex, posterolateral corners convex.

Face, clypeus, mandibles, pronotum and mesonotum smooth and shiny with scattered punctures and widely spaced foveae, each punctum and fovea with small hair. Gena with longitudinal striations and weakly marked reticulations on frontal lobes, mandibles with weak rugulae laterally near the bases. Sculpture on mesosoma consisting of transverse striations at metapleuron, rugosities at the junctions of pronotum, anepisternum, katepisternum and metapleuron, and weak rugae on dorsopropodeum. Petiole node and postpetiole dorsally smooth and shiny, lateroventrally finely areolate. Gaster smooth and shiny, foveolate near the anterodorsal corner, and with weak longitudinal carinae at articulation to postpetiole. Head and body with very short and appressed to subdecumbent pilosity. Scape and tibiae with abundant and appressed pilosity. Color light to reddish brown, gaster, legs and antennae orange to very light brown.

#### Description of minor workers.

Measurements (n=5): HW 0.33–0.41 (0.38), HL 0.37–0.45 (0.42), SL 0.26–0.29 (0.27), MDL 0.26–0.27 (0.26), EL 0.02–0.02 (0.02), WL 0.38–0.56 (0.45), PNH 0.16–0.20 (0.18), PNW 0.20–0.24 (0.22), MNH 0.22–0.27 (0.25), PDH 0.17–0.20 (0.19), PTL 0.17–0.20 (0.19), PPL 0.12–0.15 (0.14), PTH 0.12–0.14 (0.13), PPH 0.08–0.09 (0.09), PTW 0.05–0.07 (0.07), PPW 0.08–0.11 (0.10), PSL 0.05–0.06 (0.05), MFL 0.26–0.32 (0.30), MTL 0.20–0.26 (0.24), CI 88–92 (90), SI 69–84 (72), MDI 65–82 (70), EI 5–6 (5), FI 76–81 (79), PSLI 11–16 (13), LPpI 165–167 (165), DPpI 67–75 (71), PpWI 142–161 (156), PpLI 72–77 (75), PpHI 63–67 (66).

Head longer than wide (CI 88–92), in full-face view weakly subrectangular, with convex sides, nearly straight posterior head margin and rounded posterolateral corners. Mandibles with five teeth, apical and preapical teeth larger than others. Anterior margin of clypeus medially concave, bicarinate, with a small triangular tooth laterally. Frontal carinae extending beyond midlength of head. Antennae with nine segments, scapes ending before posterior quarter of head (SI 69–84). Eyes present, consisting of one ommatidium (EI 5–6).

In profile, promesonotum weakly convex, slightly more convex posterodorsally, metanotal groove narrow, but deeply impressed. Dorsum of propodeum straight to slightly convex and much shorter than posterior declivity, rounded anteriorly, declining posteriorly towards posterodorsal corners, spines small, acute-triangular, and upwardly directed, declivity of propodeum nearly straight and lamella of propodeal declivity concave medially between the spines and propodeal lobes. Propodeal spiracle situated close to posterolateral border of propodeum.

Petiole in profile with moderately short peduncle, ventral face almost straight or weakly concave at center, anteriorly directed subpetiolar process present, posteroventrally with a pronounced convex corner, petiole dorsum anteriorly concave, petiole node roundly convex. Postpetiole longer than high (LPpI 165-167), lower than petiole (PpHI 63–67), in profile dorsally convex and rounded, ventrally weakly convex. In dorsal view petiole node almost as wide as long, postpetiole almost 1.6 times wider than petiole (PpWI 142–161) with sides tapering anteriorly.

Head smooth and shiny at frons, rugulose at the sides and with scattered punctations except medially on frons, posterior margin of head with carina. Promesonotum, posterior declivity of propodeum, dorsum of petiole and postpetiole, and gaster smooth and shiny, remainder of mesosoma, petiole and postpetiole areolate rugulose. Head, scapes and tibiae with short, decumbent pilosity, mesosoma and metasoma with moderately long subdecumbent to suberect hairs and scattered shorter, decumbent pilosity. Color orange with lighter legs.

#### Distribution and biology.

*Carebara madibai* is found in the Central African Republic, Dem. Rep. Congo, Gabon and Uganda, where it was collected from leaf-litter at elevations ranging from 375–680 m. Individuals and nest series were collected from sifted litter, leaf mold and rotten wood.

#### Comments.

*Carebara madibai* is the only species of this group with nine-segmented antennae.

#### Material examined.

**CENTRAL AFRICAN REPUBLIC:** Parc National Dzanga-Ndoki, Mabéa Bai, 21.4 km 53° NE Bayanga, 3.03333, 16.41, 510m, rainforest, 10–17.v.2001 (*B.L. Fisher*); Réserve Spéciale de Forêt Dense de Dzanga Sangha, 12.7 km 326°, 3.005, 16.19333, 420m, rainforest, 10–17.v.2001, (*B.L. Fisher*); **GABON:** Woleu Ntem: 31.3 km 108° ESE Minvoul, 2.08, 12.40667, 600m, rainforest 11.ii.1998 (*B.L. Fisher*); Ogooue-Maritime, Aire d'Exploit. Rationnelle de Faune des Monts Doudou, 24.3 km 307° NW Doussala, -2.22639, 10.40972, 375m, rainforest, 6.iii.2000 (*B.L. Fisher*); **UGANDA:** Semuliki NP, 0.84483, 30.15052, 680m, rainforest, 02.viii.2012 (*B.L. Fisher* et al.).

#### Etymology.

Named in memory Nelson Rolihlahla Mandela (18 July 1918–5 December 2013), who was nicknamed Madiba by his people, former South African president and anti-apartheid revolutionary, often also described as “father of the nation”.

### Carebara nicotianae

Taxon classificationAnimaliaHymenopteraFormicidae

(Arnold) comb. n., stat. rev.

Figure 12

Sporocleptes nicotianae Arnold, 1948: 220. Three syntype(?) minor workers: ZIMBABWE: Macheke, 3.x.1948? [not examined].
Aneleus politus nicotianae (Arnold): as subspecies of *Aneleus politus* (Santschi): Arnold 1952: 460 (combination; change of status).
Oligomyrmex politus nicotianae (Arnold): Ettershank 1966: 124 (combination).

#### Diagnosis.

Antennae with 11 segments. **Major worker:** Head in full-face view wider than long, wider posteriorly than anteriorly, posterior margin of head widely and shallowly concave to V-shaped medially, face with coarse, irregular, longitudinal rugose-reticulate sculpture and weakly punctate interspaces, dorsum of scutum in profile strongly convex, propodeal spines acute, triangular and directed upward, petiole with short, anteriorly directed tooth anteroventrally, gaster with abundant, short, appressed pilosity plus longer suberect standing hairs. **Minor worker:** Head in full-face view oval with strongly convex sides, face smooth and shiny with frontal carinae ending at about cephalic midlength, posterior margin of head deeply concave, in profile propodeal dorsum straight, mesonotum and propodeum with areolate sculpture and spines well developed, moderately long and triangular. Gaster with several relatively long suberect hairs.

**Figure 12.**
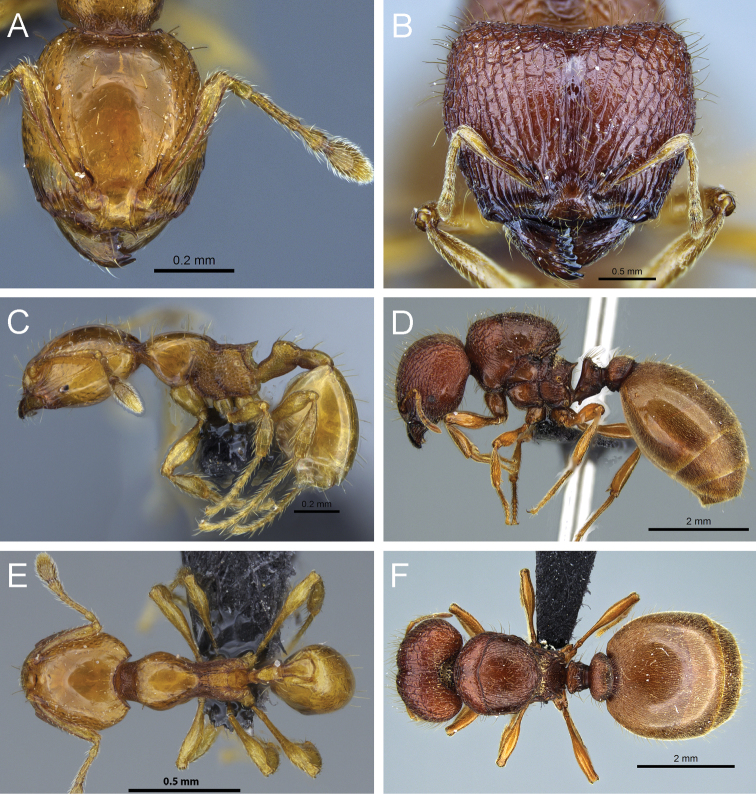
*Carebara nicotianae*. Minor worker, CASENT0066894: **A** head in full-face view **C** body in profile **E** body in dorsal view. Major worker, CASENT0066710: **B** head in full-face view **D** body in profile **F** body in dorsal view.

#### Description of major worker.

Measurements (n=1): HW 2.09, HL 1.78, SL 0.82, MDL 0.94, EL 0.15, WL 2.27, PNH 1.48, PNW 1.71, MNH 2.27, PDH 0.94, PTL 0.91, PPL 0.59, PTH 0.73, PPH 0.70, PTW 0.70, PPW 1.01, PSL 0.45, MFL 1.45, MTL 1.22, CI 117, SI 39, MDI 45, EI 7, FI 70, PSLI 21, LPpI 83, DPpI 172, PpWI 143, PpLI 64, PpHI 97.

Head distinctly wider than long (CI 117), in full-face view massive and weakly heart shaped, posteriorly wider than anteriorly. Posterior margin of head widely and shallowly emarginate, sides convex. Mandibles triangular and masticatory margin with six teeth including the basal tooth. Anterior margin of clypeus medially straight, laterally concave. Antennae eleven-segmented, scapes reaching slightly beyond midlength of head (SI 39). Eyes relatively small, multi-faceted (EI 7).

In profile, mesosoma queenlike and large, pronotum high, with large and convex scutum, scutellum inconspicuous, most likely fused with scutum. Promesonotal suture present, metanotal sclerite strongly reduced, present as narrow band below overhanging scutum-scutellum. Anepisternum and katepisternum large and well-separated from each other and segmental figs surrounded by relatively wide grooves with several short, parallel, coarse and transverse carinae. Dorsal face of propodeum nearly straight, propodeal spines relatively long, acute and with blunt tips, posterior declivity of propodeum slightly concave with a shallow lamella starting from below the base of the spines towards propodeal lobes. Propodeal spiracle situated nearly in the center of lateropropodeum.

Petiole with short peduncle, in profile ventrally straight, with rounded posteroventral corner, subpetiolar process short and digitiform, pointing anteroventrally, anterodorsal face slightly concave, posterodorsal face vertical and concave, petiole node massive and tapering apically, widely transverse on dorsal edge and posteriorly marginate. Postpetiole roundly convex in profile, about as high as petiole (PpHI 97) and distinctly higher than long (PpLI 64), convex anterodorsally, the posterior margin towards gaster oblique and linear, and with a very short ventral face. In dorsal view, petiole node anteriorly and posteriorly compressed, wider than long, anterior and posterior faces nearly straight, postpetiole about 1.4 times wider than petiole (PpWI 143), anterior and posterior faces convex.

Mandibles smooth and shiny, with weak rugulae laterally near the bases. Clypeus smooth and shiny medially, with lateral carinae. Frons and remainder of face with coarse, irregular, longitudinal rugose-reticulate sculpture, the interspaces superficially reticulate-punctate. Frontal carinae absent or inconspicuous. Dorsal pronotum anteriorly rugose-reticulate, grading to posteriorly weakly punctate, scutum grading from medially superficially punctate to laterally irregularly and posteriorly coarsely rugose-reticulate. Lateral pronotum, anepisternum and katepisternum mostly smooth and shiny, the latter weakly rugulose at margins. In profile propodeum longitudinally rugulose near the lateral base, dorsolaterally with coarse rugae and punctures in between. Posterior propodeal declivity weakly punctate. Petiole node punctate and with strong vertical rugae near the posterodorsal base. Postpetiole dorsum with irregular, longitudinal rugoreticulate sculpture. Gaster, near articulation to postpetiole, with many short, but strongly developed, longitudinal carinae, covered with a dense coat of short pubescence, and anterior quarter of first gastral tergite with faint longitudinal rugulae present.

Head and body with abundant long, erect to subdecumbent hairs, and with short, appressed to decumbent, very fine pilosity, the latter very abundant on postpetiole and gaster. Scapes and tibiae with short, very fine, and decumbent pilosity. Color reddish brown, antennae, legs and gaster yellowish light brown.

#### Description of minor workers.

Measurements (n=5): HW 0.46–0.47 (0.46), HL 0.52–0.54 (0.53), SL 0.35–0.37 (0.36), MDL 0.27–0.29 (0.28), EL 0.02–0.03 (0.02), WL 0.52–0.65 (0.56), PNH 0.23–0.24 (0.23), PNW 0.26–0.28 (0.27), MNH 0.31–0.33 (0.32), PDH 0.23, PTL 0.23–0.26 (0.24), PPL 0.14–0.15 (0.15), PTH 0.14–0.15 (0.15), PPH 0.09–0.10 (0.10), PTW 0.07–0.09 (0.08), PPW 0.11–0.12 (0.11), PSL 0.09–0.10 (0.09), MFL 0.38–0.43 (0.41), MTL 0.30–0.34 (0.31), CI 86–89 (88), SI 76–80 (77), MDI 58–61 (60), EI 5–6 (5), FI 84–90 (88), PSLI 19–21 (20), LPpI 141–167 (153), DPpI 70–81 (76), PpWI 127–161 (140), PpLI 56–67 (62), PpHI 63–68 (65).

Head longer than wide (CI 86–89), narrowed anteriorly and posteriorly, posterior margin of head sharply concave, occipital corners convex, sides strongly convex. Mandibles with five well-defined teeth. Anteromedian margin of clypeus concave, with triangular denticle laterally. Antennae with eleven segments, scapes reaching about 6/7 towards posterior margin of head (SI 76–80). Eyes consisting of one ommatidium (EI 5–6).

In profile, promesonotum weakly convex to almost straight, roundly convex at posterodorsal corner, metanotal groove weakly impressed to inconspicuous. Pronotum in profile about as high as propodeum. Dorsum of propodeum slightly convex and much shorter than posterior declivity, propodeal spines long, very acute and lamellate, lamella reaching from spines down toward propodeal lobes. Propodeal spiracle in profile close to posterior border of propodeum, somewhat below base of spine.

In profile, petiole with relatively long peduncle, ventrally weakly concave near the anterior corner and convex near the posterior corner, with a very short and angular tooth anteriorly, anterodorsally deeply concave at center, peduncle about as long as petiole node, dorsal face of node subangulate, anterodorsally rounded. Postpetiole in profile on average about 1.5 times longer than high (LpPI 141–167) and lower than petiole (PpHI 63–68), dorsally convex, bluntly angulate anteriorly and slightly rounded posteriorly, with a small, convex ventral process anteriorly. In dorsal view, petiole node about 1.5 times longer than wide, postpetiole on average 1.4 times wider than petiole (PpWI 127–161), with anteriorly converging sides. Gaster very slender in dorsal view, with acute anterior corners lateral of postpetiole attachment.

Mandibles, clypeus and face smooth and shiny, malar area up to eye level weakly longitudinally striate, with one or a few faint striae reaching posteriorly towards posterolateral lobes. Frontal carinae moderately long, continuing behind eye level to about midlength of head. Promesonotum and posterior declivity of propodeum mostly smooth and shiny, but mesopleuron and lateral and dorsal propodeum entirely areolate. Petiole areolate. Postpetiole mostly smooth and shiny and covered with weak punctures. Gaster smooth and shiny.

Head and body with several, relativel uniformly spaced, moderately long suberect to subdecumbent hairs, short decumbent pilosity very scattered and almost entirely absent from head and body surface. Posterolateral head corners with two to three short stiff hairs. Scapes and tibiae with short, decumbent pilosity. Color dark orange with slightly lighter legs and gaster.

#### Distribution and biology.

*Carebara nicotianae* is found in Zambia and Zimbabwe (type locality), collected in miombo woodland (Zambia) and at elevations ranging from 1300 to 1650 m.

#### Comments.

*Carebara nicotianae* may be confused with *Carebara polita* but major workers of *Carebara nicotianae* are without horns at posterolateral lobes while those of *Carebara polita* have small protuberances or horns, and mesonotum and propodeum of *Carebara nicotianae* minor workers are areolate while those of *Carebara polita* are smooth and shiny.

As the type material for this species could not be obtained during our studies, we used the original description and drawing of the type specimen by Arnold for the identification of our material. The facts that the material collected in Zambia is fairly close to the locality of the old types and that it does not fit the character distribution found within material of *Carebara polita* led us to the conclusion that the new material is conspecific with *Carebara nicotianae*. Therefore it is here reinstated to the status of full species.

#### Material examined.

**ZAMBIA:** Central: Lusaka, Leopard Hill, Kapuka Farm, -12.55483, 30.29567, 1300m, miombo woodland, 29.xi–3.xii.2005 (*B.L. Fisher* et al.); Central, Lusaka, Leopard Hill, Kapuka Farm, 1330m, miombo woodland, 3.xii.2005 (*B.L. Fisher* et al.); Northern, 5.3 km 247° Senga Hill, -9.386, 31.19683, 1650m, miombo woodland, 27.xi.2005 (*B.L. Fisher* et al.).

### Carebara perpusilla

Taxon classificationAnimaliaHymenopteraFormicidae

(Emery)

Figure 13

Pheidologeton perpusillum Emery, 1895: 26. Lectotype (major worker, MSNG: ANTC24569/ CASENT0904661) [designated here]: SOUTH AFRICA: Gauteng, Pretoria (*Simon*) [examined].
Carebara (Aneleus) pupusilla (Emery): Emery 1900: 327 (combination).
Aneleus perpusillus (Emery): Santschi 1914a: 77 (combination).
Oligomyrmex pupusillum (Emery): Ettershank 1966: 124 (combination).
Carebara pupusilla (Emery): Fernández 2004: 235 (combination).
Pheidologeton (Aneleus) perpusillus spinosus Forel, 1907: 17. Syntype (1 major worker, MHNG: CASENT0908885) TANZANIA: Kibosho (*Katona*) [examined]. syn. n.
Aneleus perpusillus concedens Santschi, 1914a: 77. Syntypes (1 major & 1 minor worker, NHMB: CASENT0913515, CASENT0913516) TANZANIA: Moshi, 800 m, St. 72, April 1912 (*Alluaud & Jeannel*) [examined]. syn. n.
Oligomyrmex perpusillus arnoldianus Ettershank, 1966: 123 (replacement name for *Pheidologeton perpusillum* subsp. *arnoldi* Forel, 1914: 242. Junior secondary homonym of *Oligomyrmex arnoldi* Forel, 1913a: 123). Syntype (1 major worker, BMNH: ANTC21808/ CASENT0902378): ZIMBABWE: Bulawayo, 1.iv.1913 (*G. Arnold*) [examined]. syn. n.

#### Diagnosis.

Antennae with eleven segments. **Major worker:** Head in full-face view distincly longer than wide, almost rectangular with subparallel sides and rounded posterolateral corners, a transverse carina present near posterior head margin, sculpture on mesosoma reduced, mostly consisting of weak to superficial areolae on katepisternum and lateropropodeum. **Minor worker:** Head in full-face view subrectangular, with weakly convex sides, face and pronotum smooth and shiny, posterior head margin weakly concave medially, posterolateral corners rounded.

**Figure 13.**
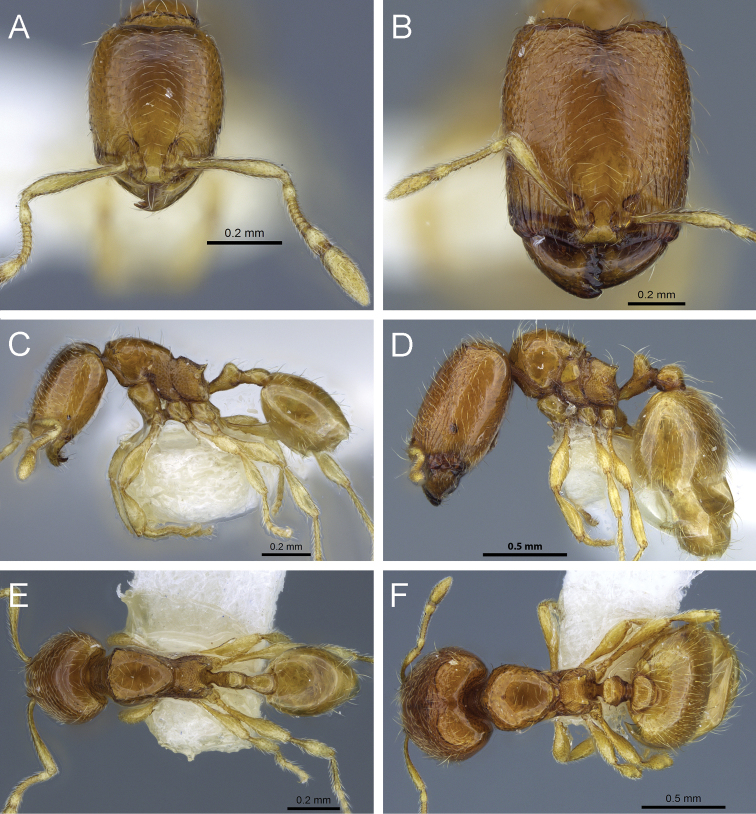
*Carebara perpusilla*. Minor worker, CASENT0914219: **A** head in full-face view **C** body in profile **E** body in dorsal view. Major worker, CASENT0914217: **B** head in full-face view **D** body in profile **F** body in dorsal view.

#### Description of major workers.

Measurements (n=5): HW 0.52–0.70 (0.62), HL 0.68–0.95 (0.80), SL 0.28–0.38 (0.32), MDL 0.32–0.49 (0.38), EL 0.0–0.05 (0.03), WL 0.53–0.69 (0.62), PNH 0.24–0.35 (0.31), PNW 0.29–0.39 (0.36), MNH 0.37–0.51 (0.44), PDH 0.23–0.29 (0.26), PTL 0.22–0.30 (0.26), PPL 0.14–0.17 (0.15), PTH 0.16–0.22 (0.19), PPH 0.14–0.18 (0.16), PTW 0.13–0.18 (0.16), PPW 0.18–0.24 (0.22), PSL 0.07–0.11 (0.09), MFL 0.32–0.44 (0.38), MTL 0.27–0.40 (0.32), CI 74–80 (77), SI 48–54 (52), MDI 56–69 (62), EI 0–7 (5), FI 57–63 (61), PSLI 14–16 (15), LPpI 88–95 (92), DPpI 132–167 (151), PpWI 128–163 (142), PpLI 50–65 (58), PpHI 81–90 (85).

Head longer than wide (CI 74–80), in full-face view nearly rectangular. Posterior margin of head roundly concave in middle, posterolateral corners slightly rounded or truncate, sides of the head weakly convex, nearly straight. Mandibles with six teeth. Anterior margin of clypeus concave in middle, sides angulate. Antennae eleven-segmented, scapes short, not surpassing cephalic midlength (SI 48–54). Eyes present, usually consisting of six to eight ommantidia (EI 0–7).

In profile, promesonotum roundly convex and higher than propodeum. Scutum and scutellum fused, promesonotal suture absent dorsally, metanotum small but separated from promesonotum. Dorsal face of propodeum straight, or slightly concave, declining posteriorly, propodeal spines subtriangular, directed slightly forward, posterior declivity of propodeum concave in profile. Propodeal spiracle roundly convex, situated closer to dorsum of propodeum than middle, lamella present but not well-developed.

Petiole with long peduncle, in profile ventrally nearly straight or weakly convex, anterior subpetiolar process present as small tooth, petiole node relatively high, dorsally weakly rounded and anteriorly and posteriorly relatively straight to slightly convex. Postpetiole in profile dorsally roundly convex, lower than petiole. In dorsal view petiole node wider than long, anteroposteriorly flattened and slightly convex, sides somewhat rounded, postpetiole about 1.5 times wider than long (DPpI 132–167), on average 1.4 times wider than petiole (PpWI 128–163), shaped almost as a half-circle, with weakly concave anterior, and convex lateral and posterior faces.

Mandibles, clypeus and face mostly smooth and shiny with scattered punctures, head laterally and posteriorly with fine or well-defined, irregular striations or reticulations, gena with well developed and longitudinal reticulations extending to eye level, weakly marked reticulations present on frontal lobes, frontal carinae absent or inconspicuous. Dorsom of promesonotum smooth and shiny, remainder of mesosoma areolate-rugose to weakly areolate, but sculpture on antero- and lateropronotum and on anepisternum often effaced to partly smooth. Petiole node and postpetiole dorsally smooth and shiny, lateroventrally finely areolate. Gaster smooth and shiny with scattered punctures on dorsum.

Head and body with moderately long, suberect standing hairs and fine, decumbent to subdecumbent pilosity, both hair types abundant on gaster. Scapes and tibiae with abundant, decumbent pilosity. Color reddish brown, legs and antennae yellowish.

#### Description of minor workers.

Measurements (n=7): HW 0.31–0.39 (0.35), HL 0.37–0.46 (0.42), SL 0.24–0.30 (0.28), MDL 0.20–0.27 (0.23), EL 0.01–0.02 (0.02), WL 0.38–0.56 (0.44), PNH 0.14–0.20 (0.17), PNW 0.20–0.25 (0.23), MNH 0.21–0.28 (0.25), PDH 0.15–0.20 (0.17), PTL 0.14–0.18 (0.15), PPL 0.09–0.11 (0.10), PTH 0.09–0.10 (0.11), PPH 0.06–0.08 (0.07), PTW 0.05–0.09 (0.07), PPW 0.08–0.11 (0.09), PSL 0.05–0.07 (0.06), MFL 0.25–0.32 (0.28), MTL 0.20–0.27 (0.24), CI 83–87 (85), SI 77–79 (78), MDI 63–69 (65), EI 4–6 (5), FI 77–81 (80), PSLI 14–19 (17), LPpI 133–173 (153), DPpI 81–93 (87), PpWI 122–143 (132), PpLI 59–71 (66), PpHI 60–67 (64).

Head longer than wide (CI 83–87), in full-face view weakly subrectangular, with convex sides, posterior margin slightly concave in middle, posterolateral corners rounded. Mandibles with five teeth, apical and preapical teeth larger than the rest. Anterior margin of clypeus nearly straight, or weakly concave, bicarinate. Frontal carinae not reaching midlength of head, ending posterior of eye level. Antennae with eleven segments, scapes ending before posterior margin of head (SI 77–79). Eyes consisting of one ommatidium (EI 4–6).

In profile, promesonotum weakly convex, anterodorsally marginate, metanotal groove rounded and deeply impressed. Dorsum of propodeum straight, declining towards posterodorsal corners, and about as long as weakly concave posterior declivity, spines acute-triangular and upwardly directed. Propodeal spiracle rounded and situated closely beneath the base of spines.

Peduncle of petiole about as long as petiole node, in profile ventral face anteriorly weakly concave, with very short to almost inconspicuous angulate tooth, and posteriorly convex, petiole node low and dorsally rounded to subtriangular. Postpetiole in profile on average 1.5 times longer than high (LPpI 133-173), lower than petiole (PpHI 60-67), weakly convex dorsally and ventrally weakly concave. In dorsal view, petiole node slightly wider than long, weakly convex laterally, anteriorly and posteriorly slightly convex, postpetiole on average 1.3 times wider than petiole (PpWI 122-143), posterior face roundly convex, with sides oblique and tapering anteriorly.

Mandibles, clypeus and face smooth and shiny with relatively abundant punctures. Promesonotum smooth and shiny, remainder of mesosoma areolate. Petiole and postpetiole areolate–rugose, except for smooth and shiny dorsum of petiole node and lateral and dorsal face of postpetiole. Gaster smooth and shiny.

Head and body with relatively few erect to suberect standing hairs and relativley abundant, decumbent pilosity. Scapes and tibiae with decumbent pilosity. Color yellowish orange.

#### Distribution and biology.

*Carebara perpusilla* is a widespread species found in Kenya, Rwanda, South Africa, Tanzania, Uganda, Zambia, and Zimbabwe, mainly in primary rainforest (Kenya) and miombo woodland (Zambia). *Carebara perpusilla* has been collected at elevations ranging from 42–2100 m. Individuals and nest series were collected in leaf-litter and from soil, using Winkler, pitfall and pan traps.

#### Comments.

*Carebara perpusilla* is similar to, but smaller than *Carebara silvestrii*. Also, major workers of *Carebara perpusilla* have a smooth and shiny lateropronotum and anepisternum, while these parts are densely areolate in *Carebara silvestrii*. The minor workers of *Carebara perpusilla* have a smooth and shiny face and pronotum, while in *Carebara silvestrii* these parts are never completely smooth, and usually have some areolate or rugose sculpture.

#### Material examined.

**KENYA:** Western, Kakamega Forest, Salazar, 0.32667, 34.87083, 1650m, primary forest habitat, 9.iii.2009 (*Marcell Peters*); Central Province, Mt. Kenya, Chogoria, point D, 24 km from gate, 3171m, 9–12.iii.2009 (*J. Mugambi*); Coastal Province, Arabuko Sokoke Forest, 3.27257, 39.91735, 42m, Cynometra forest, vi.2009 (*G. Fischer* & F. *Hita Garcia*); Kakamega district, Isechen Nature Reserve, nr Kalunya Glade, 0.24, 34.86, 1800m, 4.v.2001, rainforest (*R. R. Snelling*). Kenya: Lamu, nr Witu, 26.x.1977 (*V. Mahnert* & *J.L. Perret*); Tana Riv. Galole, 60m, 21.x.1977 (*V. Mahnert* & *J. L. Perret*); Tana Riv, Wema, 24.x.1977 (*V. Mahnert* & *J.L. Perret*); **RWANDA:** Rangiro, ix.1976 (*P. Werner*); **SOUTH AFRICA:** KwaZulu-Natal, Umtamvuna Nature Reserve, -31.04507, 30.168, 220m, Pondoland coastal figau, sour grassland, 18.xi.2000 (*S. Van Noort*); KwaZulu-Natal, Umtamvuna Nature Reserve, -31.04507, 30.168, 220m, Pondoland coastal figau, sour grassland, 10–17.xi.2000 (*S. Van Noort*); **UGANDA:** Ruwenzori, above ibanda, 2100m, 9.v.1993 (*Cuccodoro* & *Erne*); Mt Elgon, Sipi, 1750m, 31.v.1993 (*Cuccodoro* & *Erne*); **ZAMBIA:** Central, Lusaka, Leopard Hill, Kapuka Farm, -12.55483, 30.29567, 1300m, miombo woodland, 2.xii.2005 (*B.L. Fisher* et al.).

### Carebara polita

Taxon classificationAnimaliaHymenopteraFormicidae

(Santschi)

Figure 14

Aneleus politus Santschi, 1914a: 79. Syntypes (3 minor workers, CASENT0913517) KENYA: Blue Post Hotel (Thika, ca. 40 km NE of Nairobi), 1520m, i.1912 (*Alluaud* & *Jeannel*) [3 syntypes examined].
Oligomyrmex politus (Santschi): Ettershank 1966: 124 (combination).
Carebara polita (Santschi): Fernández 2004: 235 (combination).

#### Diagnosis.

Antennae with 11 segments. **Major worker:** Head in full-face view about as long as wide, posterior wider than anterior, posterior head margin with widely V-shaped emargination and small, tooth-like horns at posterolateral corners, propodeum with short, triangular, bluntly rounded spines, dorsum of propodeum nearly straight or very weakly convex, petiole with small, shallowly triangular ventral process, gaster with little, very short and appressed pilosity. **Minor worker:** Head in full-face view oval with strongly convex sides and deeply concave posterior emargination, face and most of mesosoma smooth and shiny, frontal carinae not extending beyond cephalic midlength, spines elongate-triangular, in dorsal view about as long as distance between them, gaster smooth and shiny and with or without very few standing hairs.

**Figure 14.**
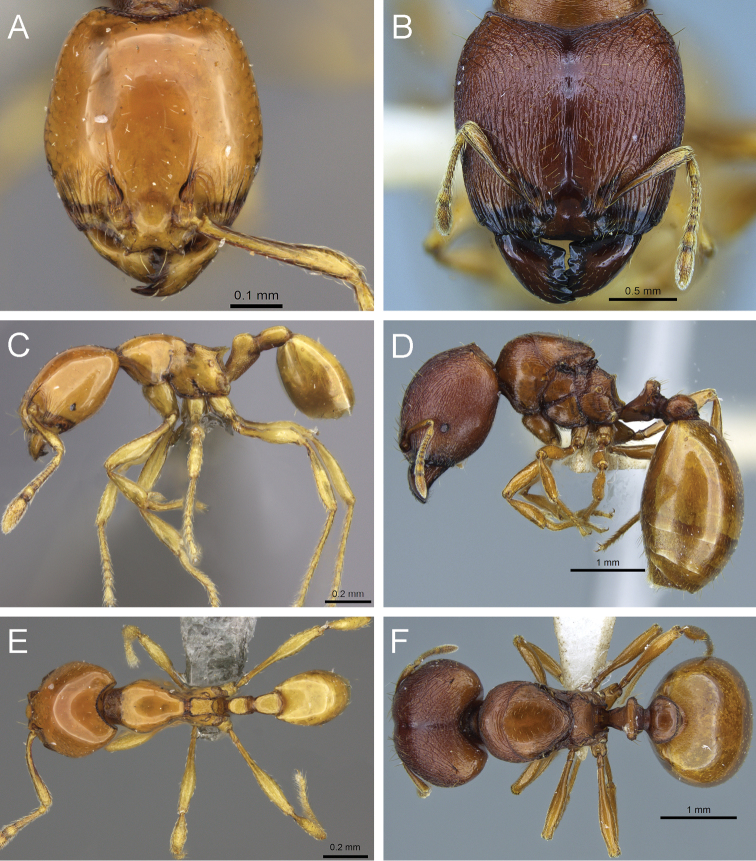
*Carebara polita*. Minor worker, CASENT0235574: **A** head in full-face view **C** body in profile **E** body in dorsal view. Major worker, CASENT0914213: **B** head in full-face view **D** body in profile **F** body in dorsal view.

#### Description of major workers.

Measurements (n=6): HW 1.20–1.74 (1.62), HL 1.17–1.77 (1.59), SL 0.65–0.74 (0.70), MDL 0.68–0.86 (0.80), EL 0.09–0.15 (0.13), WL 1.58–1.80 (1.71), PNH 0.83–1.05 (0.97), PNW 0.93–1.17 (1.07), MNH 1.13–1.35 (1.28), PDH 0.72–0.77 (0.75), PTL 0.65–0.72 (0.68), PPL 0.42–0.48 (0.45), PTH 0.54–0.57 (0.55), PPH 0.47–0.51 (0.48), PTW 0.47–0.58 (0.55), PPW 0.63–0.69 (0.67), PSL 0.29–0.33 (0.31), MFL 1.07–1.16 (1.12), MTL 0.84–0.95 (0.89), CI 97–108 (102), SI 40–54 (44), MDI 45–56 (50), EI 5–9 (8), FI 62–94 (71), PSLI 17–28 (19), LPpI 88–100 (94), DPpI 140–153 (148), PpWI 109–135 (122), PpLI 63–70 (60), PpHI 84–89 (87).

Head about as long as wide (CI 97–108), in full-face view weakly subquadrate, with posteriorly slightly diverging, convex sides, posterior margin usually triangularly emarginate and with small protuberances or horns at posterolateral lobes. Mandibles with five well-developed teeth. Clypeus slightly concave at anteromedian margin, laterally with two carinae. Antennae eleven-segmented, scapes short, reaching slightly beyond midlength of head (SI 40–54). Eyes present, with between ten and sixteen ommatidia (EI 5–9).

In profile, promesonotum roundly convex, much higher than propodeum. Scutum well-developed, scutellum either fused with scutum or vestigial in most specimens, but well-developed in a few major workers. Promesonotal suture present on dorsum, metanotum developed and usually well-separated. Anepisternum and katepisternum large and well separated by a wide groove with some cross-ribs. Dorsal face of propodeum straight and declining posteriorly, propodeal spines comparatively short and stout with rounded tips, declivity of propodeum slightly concave, with lamella reaching from basal edge of spines towards propodeal lobes. Propodeal spiracle close to center of propodeum.

Petiole with moderately short peduncle, in profile ventrally weakly concave with rounded posterior corner, subpetiolar process short and subtriangular to reduced, petiole node in profile high and subrectangular, anteriorly concave, posterior margin vertical and nearly straight, dorsal face nearly straight or weakly convex. Postpetiole in profile lower than petiole (PpHI 84–89), subglobular, almost as long as high (LpPI 88–100), dorsally roundly convex, ventral face much shorter than dorsal face. In dorsal view, petiole wider than long, strongly flattened anteroposteriorly, anterior and posterior faces straight to almost straight, postpetiole weakly hexagonally shaped, on average 1.2 times as wide as petiole (PpWI 109–135), and sides weakly subangulate anteriorly and towards posterior face.

Mandible and head with scattered punctures, except medially on frons. Mandibles smooth and shiny, and laterally weakly striate, clypeus smooth and shiny, with lateral carinae, frons finely longitudinally rugulose, rugulae diverging posteriorly towards posterolateral lobes, at sides of head weaker and more irregular, interspaces weakly to faintly punctate-rugose. Frontal carinae weakly developed, length varying from reaching towards anterior eye level to about midlength of head.

Dorsum of pronotum and scutum variably circularly striate, sometimes only on margins, with center being smooth and shiny, lateral portions of pronotum, anepisternum and katepisternum mostly smooth and shiny to faintly striate. Propodeum longitudinally striate lateroventrally, dorsally and dorsolaterally coarsely sculptured with irregular rugulae, posterior propodeal declivity shagreened or micropunctate. Petiole and postpetiole densely punctate lateroventrally, grading to smooth and shiny on dorsal faces. Gaster smooth and shiny, anteriorly, at articulation with postpetiole, weakly punctate.

In profile, head and body with several short, suberect hairs and scattered short, appressed pilosity. Head in full-face view with two or three relatively coarse suberect hairs present on posterolateral lobes. Scapes and tibiae with abundant appressed and yellow pilosity. Color reddish brown, legs and antennae yellowish or light brown.

#### Description of minor workers.

Measurements (n=11): HW 0.43–0.54 (0.51), HL 0.50–0.62 (0.57), SL 0.36–0.44 (0.41), MDL 0.25–0.32 (0.30), EL 0.00–0.03 (0.02), WL 0.48–0.76 (0.58), PNH 0.23–0.29 (0.26), PNW 0.26–0.32 (0.29), MNH 0.33–0.42 (0.38), PDH 0.23–0.29 (0.26), PTL 0.23–0.29 (0.25), PPL 0.14–0.18 (0.16), PTH 0.15–0.17 (0.16), PPH 0.10–0.12 (0.11), PTW 0.08–0.12 (0.10), PPW 0.11–0.15 (0.13), PSL 0.08–0.11 (0.09), MFL 0.38–0.49 (0.45), MTL 0.29–0.38 (0.35), CI 86–91 (88), SI 78–84 (81), MDI 53–62 (59), EI 0–7 (5), FI 78–92 (88), PSLI 16–21 (18), LPpI 136–171 (147), DPpI 75–91 (83), PpWI 119–136 (126), PpLI 58–75 (63), PpHI 61–73 (66).

Head in full-face view longer than wide (CI 86–91), with strongly convex sides, posterior margin of head sharply concave, posterolateral corners angulate to subangulate. Mandibles with four well-defined teeth. Anterior margin of clypeus deeply concave medially, bicarinate and anteriorly divergent, laterally with two small, triangular teeth. Frontal carinae extending towards the cephalic midlength. Antennae with eleven segments, scapes reaching about 6/7 towards posterior margin of head (SI 78–84). Eyes usually present and consisting of one or two ommatidia, rarely no ommatidia visible (EI 0–7).

In profile, promesonotal dorsum smoothly rounded and convex, metanotal groove shallowly impressed. Dorsum of propodeum slightly convex and much shorter than posterior declivity, spines long and lamellate, lamella reaching from spines down toward large propodeal lobes. Propodeal spiracle closer to posterodorsal corner than center of propodeum.

Petiole in profile with moderately long peduncle, ventrally nearly straight, anteroventral corner with a very short to inconspicuous angulate tooth present, petiole node anteriorly slightly concave, posteriorly weakly convex, and dorsal face weakly convex to subangulate anteriorly and posteriorly. Postpetiole in profile convex, on average 1.5 times longer than high (LPpI 136–171), lower than petiole (PpHI 61–73). In dorsal view petiole anteriorly and laterally convex, postpetiole slightly wider than petiole (PpWI 119–136), postpetiole convex and tapering anteriorly, sides subparallel, posteriorly almost straight to faintly convex.

Mandibles, clypeus and face smooth and shiny, with scattered punctures, malar area up to eye level longitudinally striate. Mesosoma mostly smooth and shiny without microsculpture, propodeal lamella, dorsal and posterior propodeal margins weakly alveolate. Petiole weakly alveolate, dorsal face of node, postpetiole and gaster mostly smooth and shiny. Head and body usually without standing hairs and with very little appressed pilosity, in some specimens a few erect, relatively short and stiff hairs with blunt apices present on mesosoma and gaster. Scapes and tibiae with short, appressed pilosity. Color orange to dark brown (in Kibale and Semuliki National Parks, Uganda).

#### Distribution and biology.

*Carebara polita* is a widespread species found in Cameroon, Central African Republic, Gabon, Kenya, Tanzania, and Uganda, mainly in rainforest, montane wet forest, and evergreen forest. *Carebara polita* has been collected at elevations ranging from 110–2045 m. Individuals and nest series were collected in leaf-litter and from soil, using Winkler and pitfall traps.

#### Comments.

*Carebara polita* can be easily separated from the other species, because major workers have small protuberances or horns at the posterolateral lobes of head. Minor workers with sides of head strongly convex and head and body mostly smooth and shiny. This species can be confused with *Carebara nicotianae*, but major workers of *Carebara nicotianae* do not have horns at the posterolateral lobes, and the mesonotum and propodeum are areolate in *Carebara niconianae* minor workers, but mostly smooth and shiny in *Carebara polita*.

#### Material examined.

**CAMEROON:** Sud-Ouest, Korup N. P., 6.9 km 317° NW Mundemba, 5.016, 8.864, 110m, rainforest, 19.iv.2000 (*B.L. Fisher*); **CENTRAL AFRICAN REPUBLIC:** Prefecture Sangha-Mbaéré, Parc National Dzanga Ndoki, 38.6 km 173° S Lidjombo, 2.36, 16.14397, 350m, rainforest, 21–27.v.2001 (*S. Van Noort*); **GABON:** Ogooue-Maritime, Reserve de Faune de la Moukalaba Dougoua, 12.2 km 305° NW Doussala, -2.31667, 10.53333, 110m, rainforest, 24.ii.2000 (*B.L. Fisher*); Ogooue Maritime, Aire d'Exploit. Rationnelle de Faune des Monts Doudou, 24.5 km 303° WNW Doussala, -2.23283, 10.398, 630m, rainforest, 18.iii.2000 (*B.L. Fisher*); Woleu Ntem, 31.3 km 108° ESE Minvoul, 2.08, 12.40667, 600m, rainforest, 7.ii.1998, (B.L. Fisher); **KENYA:** Western, Kakamega Forest, Salazar, 0.32667, 34.87083, 1650m, primary forest habitat, 9.iii.2009 (*Marcell Peters*); Western, Kakamega Forest, Ivalakale farmland, 0.34556, 34.865, 1650m, farmland, 09.vii.2008 (*G. Fischer*); Western, Kakamega District, Isecheno, Isecheno Forest Reserve, 0.24, 34.86, 1600m, 31.viii.2001 (*W. Okeka*); Kikiyu. Esc. Gatamayu, 233 m, montane forest, ii.1999 (*Th. Wagner*); Mt. Kenya, 0.7km NE Kangaita Forest Sta, 2045m, montane wet forest, 23.i.2000 (*R.R. Snelling*); **TANZANIA:** Morogora, Udzungwa Mts. 4.5km W. Chita, 600m, 28.viii.1995 (*W.T. Stanley*); Morogora, Udzungwa Mts. 3.5km W. 1.7km N Chita, 910m, 7.viii.1995 (*W.T. Stanley*); **UGANDA:** Mt. Elgon, Sipi, 1750m, 01.vi.1993 (*Cuccodoro* & *Erne*); Kabarole, Kanyawara, Kibale NP, 0.56427485, 30.358759, 1520m, mature wet forest, 4–26.viii.2008 (*S. Van Noort*); Kabarole, Kanyawara, Kibale NP, 0.56427485, 30.358759, 1510m, evergreen forest, 6–16.viii.2012 (*Ant Course 2012*); Semuliki NP, 0.83556, 30.15542, 676m, rainforest, 30.vii.-1.viii.2012 (*B.L. Fisher* et al.).

### Carebara silvestrii

Taxon classificationAnimaliaHymenopteraFormicidae

(Santschi) comb. n.

Figure 15

Aneleus silvestrii Santschi, 1914b: 357. Lectotype (1 major worker, NHMB: ANTC27952/ CASENT0913522) [designated here]: GHANA: Cöte d' Or, Aburi [Abury], (*Silvestri*) [1 minor and 1 major syntype examined].
Oligomyrmex silvestrii (Arnold): Ettershank 1966: 124 (combination).
Aneleus (Aneleus) punctatus Karavaiev, 1931: 43. Holotype minor worker: KENYA: Mabira (no. 5323) [not examined] syn. n.

#### Diagnosis.

Antennae with eleven segments. **Major worker:** Head nearly rectangular or subquadrate but distincly longer than wide, with parallel to weakly convex sides, rounded posterolateral corners and transverse carina present near posterior head margin, lateral portions of mesosoma usually extensively areolate, dorsal face of head smooth and shiny in larger major workers and with sculpture in medium major workers except for medially smooth and shiny frontal area, propodeum with a pair of short, acute, subtriangular spines, gaster covered with abundant decumbent hairs. **Minor worker:** Head suboval in full-face view, sides strongly convex, posterolateral corners subangulate, and posterior margin with convex emargination, frons smooth and shiny, remainder of face with longitudinal rugulae and reticulations, propodeal spines slightly elongate triangular to short spinose, gaster with several decumbent and moderately long hairs.

**Figure 15.**
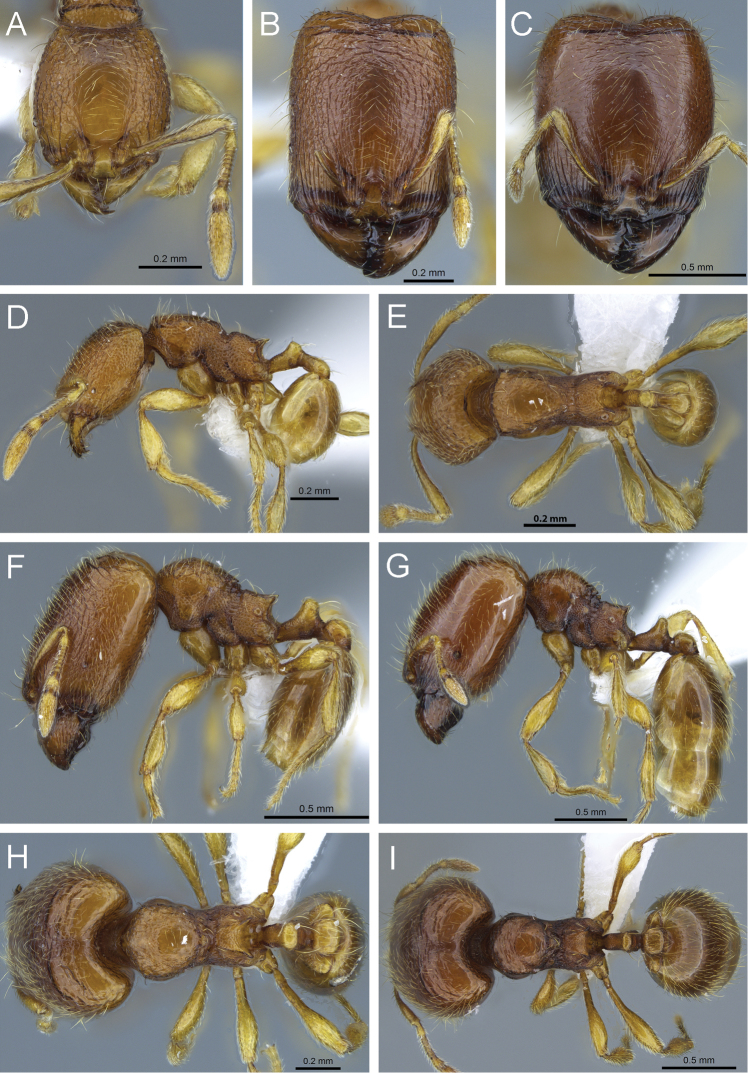
*Carebara silvestrii*. Minor worker, CASENT0914215: **A** head in full-face view **D** body in profile **E** body in dorsal view. Major worker, CASENT0914214: **B** head in full-face view **F** body in profile **H** body in dorsal view. Large major worker, CASENT0914216: **C** head in full-face view **G** body in profile **I** body in dorsal view.

#### Description of major workers.

Measurements (n=6): HW 0.64–0.99 (0.85), HL 0.79–1.15 (1.02), SL 0.34–0.45 (0.41), MDL 0.38–0.57 (0.49), EL 0.04–0.07 (0.05), WL 0.58–0.87 (0.75), PNH 0.24–0.46 (0.37), PNW 0.32–0.48 (0.42), MNH 0.39–0.62 (0.53), PDH 0.24–0.38 (0.31), PTL 0.20–0.36 (0.28), PPL 0.15–0.21 (0.18), PTH 0.17–0.27 (0.22), PPH 0.14–0.23 (0.18), PTW 0.13–0.18 (0.15), PPW 0.15–0.23 (0.20), PSL 0.09–0.15 (0.12), MFL 0.37–0.60 (0.50), MTL 0.31–0.52 (0.43), CI 76–87 (83), SI 45–53 (49), MDI 56–61 (58), EI 6–7 (6), FI 57–65 (59), PSLI 13–16 (14), LPpI 92–107 (99), DPpI 100–128 (112), PpWI 122–144 (133), PpLI 56–81 (66), PpHI 76–88 (83).

Head longer than wide (CI 76–87), in full-face view nearly rectangular to subquadrate. Posterior margin of head weakly concave to V-shaped medially, posterolateral corners roundly convex, sides of the head nearly straight to slightly convex. Mandibles triangular and masticatory margin with five teeth including the basal tooth. Frontal carinae short or inconspicuous. Anterior margin of clypeus concave medially, sides angulate. Antennae with eleven segments, scapes short and not surpassing cephalic midlength (SI 45–53). Eyes small and consisting of one ommatidium (EI 6-7).

In profile, promesonotum roundly convex and higher than propodeum. Promesonotal suture present or absent on dorsum, metanotal groove present and impressed. Dorsal face of propodeum straight in profile, or slightly concave and declining posteriorly, anterodorsal corner weakly angulate, propodeal spines stout and subtriangular, posterior declivity concave. Propodeal spiracle rounded, situated slightly above center of lateral propodeum.

Petiole with relatively long peduncle, ventrally weakly concave anteriorly and slightly convex posteriorly, sometimes nearly straight over the whole length, subpetiolar process present as a small, angulate, forward-directed tooth, petiole node relatively high, anteroposteriorly somewhat compressed, dorsally subangulate anteriorly and posteriorly in smaller major workers, rounded in larger majors. Postpetiole in profile roundly convex dorsally, about as high as long (LPpI 92–107), and lower than petiole (PpHI 76–88). In dorsal view petiole node small, wider than long, anteriorly and posteriorly flattened and nearly straight to weakly convex, sides subangulate, postpetiole slightly wider than long (DpPI 100–128), about 1.3 times wider than petiole (PpWI 122–144), anteriorly and posteriorly weakly convex, laterally roundly convex.

Mandibles, clypeus and frons medially, and in large majors most of face, smooth and shiny with scattered punctures. In small majors sides of face with irregular longitudinal reticulations, grading to transverse reticulations towards posterior head margin, and with a transverse carina near posterior margin. In large majors, posterior head margin with transverse carina and with short transverse reticulations anterior and posterior of transverse carina. In all majors gena and frontal lobes with well-defined longitudinal striations. Dorsum of promesonotum centrally smooth and shiny, weakly reticulate near its margins, remainder of mesosoma areolate to weakly areolate, sometimes locally effaced on lateropronotum and katepisternum. Petiole node and postpetiole dorsally smooth and shiny, lateroventrally finely areolate. Gaster smooth and shiny with scattered punctures.

Head and body with moderately long suberect to subdecumbent hairs and short decumbent pilosity, scapes and tibiae with mostly decumbent pilosity. Color reddish brown, legs and antennae yellowish.

#### Description of minor workers.

Measurements (n=4): HW 0.36–0.44 (0.40), HL 0.41–0.51 (0.46), SL 0.29–0.34 (0.32), MDL 0.23–0.29 (0.26), EL 0.02, WL 0.45–0.59 (0.53), PNH 0.17–0.22 (0.19), PNW 0.23–0.27 (0.25), MNH 0.25–0.31 (0.28), PDH 0.17–0.22 (0.19), PTL 0.15–0.18 (0.16), PPL 0.11–0.12 (0.11), PTH 0.09–0.11 (0.10), PPH 0.08–0.09 (0.08), PTW 0.07–0.08 (0.08), PPW 0.08–0.10 (0.09), PSL 0.07–0.08 (0.07), MFL 0.30–0.39 (0.34), MTL 0.26–0.32 (0.29), CI 86–88 (87), SI 76–91 (81), MDI 62–66 (65), EI 4–6 (5), FI 80–91 (84), PSLI 17–19 (18), LPpI 139–160 (147), DPpI 69–81 (75), PpWI 109–122 (115), PpLI 67–80 (71), PpHI 71–83 (77).

Head longer than wide (CI 86–88), in full-face view anteriorly and posteriorly narrowed, sides strongly convex, posterior margin of head sharply concave, posterolateral corners angulate. Mandibles triangular with five teeth. Anterior margin of clypeus concave, bicarinate and divergent forward, sides angulate. Frontal carinae not surpassing midlength of head. Antennae eleven-segmented, scapes not reaching posterior head margin (SI 76–91). Eyes consisting of one ommatidium (EI 4-6).

In profile, promesonotum weakly convex, posterodorsal corner roundly convex, metanotal groove rounded and deeply impressed, pronotum anterodorsally transversely carinate. Dorsum of propodeum in profile straight, declining posteriorly, anterodorsal corner rounded, propodeal spines subtriangular and upwardly directed, posterior declivity of propodeum concave, lamella extending from the spines to the lobes. Propodeal spiracle rounded and situated below the base of spines and close to posterior border of lateral propodeum.

Petiole with relatively long peduncle, ventrally weakly concave anteriorly and slightly convex posteriorly, sometimes nearly straight over whole length, subpetiolar process present as small, angulate, forward-directed tooth, petiole node relatively low, small, subtriangular, or subangulate anteriorly and posteriorly. Postpetiole roundly convex in profile, on average about 1.5 times longer than high (LPpI 139–160), and lower than petiole (PpHI 71–83). In dorsal view petiole node small, almost wider than long, anteriorly weakly convex, posteriorly almost straight, sides subangulate, postpetiole longer than wide (DpPI 69–81), slightly wider than petiole (PpWI 109–122), anteriorly tapering, almost pedunculate, sides and posterior face roundly convex.

Mandibles, clypeus and frons smooth and shiny with scattered punctures, remainder of face irregularly rugreticulate. Dorsum of promesonotum weakly reticulate except for smooth and shiny center, and central area of lateral pronotum with effaced sculpture. Remainder of lateropronotum, mesopleuron and propodeum areolate, except smooth and shiny posterior declivity. Gaster and dorsum of petiole node and postpetiole smooth and shiny, remainder weakly areolate to areolate-rugose.

Head and body with moderalely long suberect to subdecumbent hairs and short decumbent pilosity. Scapes and tibiae with decumbent pilosity. Color reddish brown, legs and antennae yellowish.

#### Distribution and biology.

*Carebara silvestrii* is a widespread species found in Cameroon, Central African Republic, Equatorial Guinea, Gabon, Ghana (type-locality), Ivory Coast, Kenya, Uganda and Zimbabwe, mainly in rainforest. *Carebara silvestrii* has been collected at elevations ranging from 10–2250 m. Individuals and nest series were collected from the leaf-litter and the soil with Winkler sifting, pitfall traps, and hand collections.

#### Comments.

*Carebara silvestrii* may be confused with *Carebara perpusilla*, but lateropronotum and anepisternum are smooth and shiny in major workers of *Carebara perpusilla* while in *Carebara silvestrii* they are areolate. The frons is often smooth and shiny in minor workers of *Carebara silvestrii* and the remainder of the face usually coarsely rugoreticulate, while the entire head is smooth and shiny in *Carebara perpusilla*. Although type specimens could not be obtained, *Carebara punctata* (Karavaiev) is synonymised with *Carebara sylvestrii* based on the details provided in the original description.

#### Material examined.

**CAMEROON:** Sud, P. N. Campo, 43.3 km 108° ESE Campo, 2.2825, 10.20617, 290m, rainforest, 7.iv.2000 (*B.L. Fisher*); Sud Ouest, Mt. Cameroon, Etinde Forest Res., 3.8 km 330° NNW Mapanja, 4.10767, 9.152, 1440m, 16.iv.2000 (*B. L. Fisher*); Sud, Res. de Faune de Campo, Massif des Mamelles, 15.1 km 84° E Ébodjé, 2.59417, 9.9595, 180m, 4.iv.2000, (*B.L. Fisher*); Sud: Bondé Forest, N'kolo village, 27.5 km 155° SSE Elogbatindi, 3.22167, 10.24667, 40m, rainforest, 12.iv.2000 (*B.L. Fisher*); **CENTRAL AFRICAN REPUBLIC:** Prefecture Sangha Mbaéré, Parc National Dzanga Ndoki, 37.9 km 169° S Lidjombo, 2.37067, 16.1725, 360m, rainforest, 21.v.2001 (*B.L. Fisher*); Prefecture SanghaMbaéré, Réserve Spéciale de Forêt Dense de Dzanga Sangha, 12.7 km 326° NW Bayanga, 3.005, 16.19333, 370m, rainforest, 10–17.v.2001 (*B. L. Fisher*); Prefecture Sangha-Mbaéré, Réserve Spéciale de Forêt Dense de Dzanga Sangha, 12.7 km 326° NW Bayanga, 3.005, 16.19333, 470m, rainforest, 10–17.v.2001 (*B. L. Fisher*); **GABON:** Ogooue-Maritime, Aire d'Exploit. Rationnelle de Faune des Monts Doudou, 24.3 km 307° NW Doussala, 2.22639, 10.40972, 375m, rainforest, 6.iii.2000 (*B.L. Fisher*); Woleu-Ntem, 31.3 km 108° ESE Minvoul, 2.08, 12.40667, 600m, rainforest, 7.ii.1998 (*B.L. Fisher*); Estuaire, F.C. Mondah, 21 km 331° NNW Libreville, 0.57667, 9.335, 10m, litoral rainforest, 24.ii.1998 (*B.L. Fisher*); Woleu-Ntem, 31.3 km 108° ESE Minvoul, 2.08, 12.40667, 600m, rainforest, 11.ii.1998 (*B.L. Fisher*); Ogooue-Maritime: Reserve de Faune de la Moukalaba-Dougoua, 12.2 km 305° NW Doussala, -2.31667, 10.53333, 110m, rainforest, 24.ii.2000 (*B.L.Fisher*); Ogooue Maritime: Aire d'Exploit. Rationnelle de Faune des Monts Doudou, 24.5 km 303° WNW Doussala, -2.23283, 10.398, 630m, rainforest, 18.iii.2000 (*B. L. Fisher*); Ogooue Maritime, Aire d'Exploit. Rationnelle de Faune des Monts Doudou, 24.3 km 307° NW Doussala, -2.22639, 10.40972, 375m, rainforest, 9.iii.2000, (*B. L. Fisher*); Ogooue-Maritime, Reserve de Faune de la Moukalaba-Dougoua, 10.8 km 214° SW Doussala, -2.42267, 10.54533, 110m, rainforest, 29.ii.2000 (*B.L. Fisher*); Ogooue-Maritime, Aire d'Exploit. Rationnelle de Faune des Monts Doudou, 25.2 km 304° NW Doussala, -2.2275, 10.3945, 640m, rainforest, 19.iii.2000 (*B. L. Fisher*); Ogooue-Maritime, Aire d'Exploit. Rationnelle de Faune des Monts Doudou, 24.3 km 307° NW Doussala, -2.22639, 10.40972, 375m, rainforest, 9.iii.2000 (*B.L. Fisher*); Ogooue-Maritime, Aire d'Exploit. Rationnelle de Faune des Monts Doudou, 25.2 km 304° NW Doussala, -2.2275, 10.3945, 640m, rainforest, 14.iii.2000 (*B.L. Fisher*); **IVORY COAST:** Mar, 900m, Mt. Tonkoni, 13.x.1980 (*V. Mahnert* & *Perret*); **KENYA:** Western, Kakamega Forest, Kaimosi Forest Fragment, 0.12806, 34.84, 1650m, primary rain forest, 27.viii.2005 (*G. Fischer*); Western, Kakamega Forest, Ikuywa, 0.21167, 34.93139, 1650 m, primary forest, 16.viii.2007 (*F. Hita Garcia*); Western, Kakamega Forest, Yala, 0.20144, 34.88073, 1650m, primary forest, v.2008 (*Marcell Peters*); Western, Kakamega Forest, Isecheno B, 0.24778, 34.86806, 1650m, primary forest, 9.ix.2008 (*Florian Herchen*); Western, Kakamega Forest, Isecheno A, 0.24944, 34.86806, 1650m, primary forest, 17.vii.2007 (*Susanne Maurer*); **UGANDA:** Mt Elgon, Kapkwata, 2250m, 30.v.1993 (*Cuccodoro* & *Erne*); Kibale Forest, 1250m, 23.v.1993 (*Cuccodoro* & *Erne*); Ruwenzori, Ibanda, 1650m, 10.v.1993 (*Cuccodoro* & *Erne*); Kibale NP, Kanyawara Biol. Stn, 0.55878025, 30.359982, 1510m, evergreen forest, 6–16.viii.2012 (*Ant Course 2012*); Bunyoro District, Budongo Forest FS, 1.72638, 31.55237, 1081m, 8.vii.2009 (*W. Freund* & *T. Klug*); **ZIMBABWE:** Umtali, Melsetter, 1700m, ii.1969 (*R. Mussard*).

### Carebara urichi

Taxon classificationAnimaliaHymenopteraFormicidae

(W.M. Wheeler)

Figure 16

Spelaeomyrmex urichi W.M. Wheeler, 1922b: 9. Syntypes (11 minor workers) TRINIDAD: “Guacharo Cave” (*F.M. Urich*) [not examined].
Erebomyrma urichi (W.M. Wheeler): Wilson 1962: 63 (combination).
Oligomyrmex urichi (W.M. Wheeler): Ettershank 1966: 124 (combination).
Carebara urichi (W.M. Wheeler): Fernández 2004: 205 (combination).
Erebomyrma nevermanni (Mann): junior synonym of *Carebara urichi*: Fernández 2004: 205. Type (1 minor worker): COSTA RICA: Hamburg Farm, Reventazon, Santa Clara (*F. Nevermann*) [not examined].
Oligomyrmex nevermanni (Mann): Ettershank 1966: 124 (combination).
Erebomyrma nevermanni (Mann): Wilson 1986: 61 (combination).
Oligomyrmex nevermanni (Mann): Bolton 1995: 299 (combination).
Erebomyrma morai (Menozzi): junior synonym of *Carebara urichi*: Fernández 2004: 205. Syntype (1 minor worker) COSTA RICA: S. Josè (*F.I. Tristan*) [BMNH] [examined].
Oligomyrmex morai (Menozzi): Ettershank 1966: 124 (combination).
Erebomyrma morai (Menozzi): Brandão, 1991: 343 (combination).
Oligomyrmex morai (Menozzi): Bolton 1995: 299 (combination).
Erebomyrma eidmanni (Eidmann): attributed to Menozzi; junior synonym of *Carebara urichi*: Fernández 2004: 205. BRAZIL: Mendes, 3.x.1933 (*Eidmann*) [types not examined].
Oligomyrmex eidmanni (Menozzi): Ettershank 1966: 123 (combination).
Erebomyrma eidmanni (Menozzi): Wilson 1986: 61 (combination).
Oligomyrmex eidmanni (Menozzi): Bolton 1995: 299 (combination).

#### Diagnosis.

Antennae with eleven segments. **Major worker:** Head almost as long as wide to slightly longer, nearly subquadrate in full-face view, posterolateral corners rounded. Head with longitudinal striations, rugulae on the posterior side, propodeal spines absent or inconspicuous, petiolar ventral process large, digitiform and anteriorly directed, gaster covered by very abundant erect hairs. **Minor worker:** Head subrectangular, sides convex, posterior margin almost straight, posterolateral corners angulate, frons medially smooth and shiny, face with longitudinal rugulae and reticulations, propodeal spines short, acute-triangular and upwardly directed, metatibia with long, suberect hairs along outer edge, petiole anteroventrally with small, anteriorly-pointing tooth, gaster with relatively abundant, long, suberect or subdecumbent hairs.

**Figure 16.**
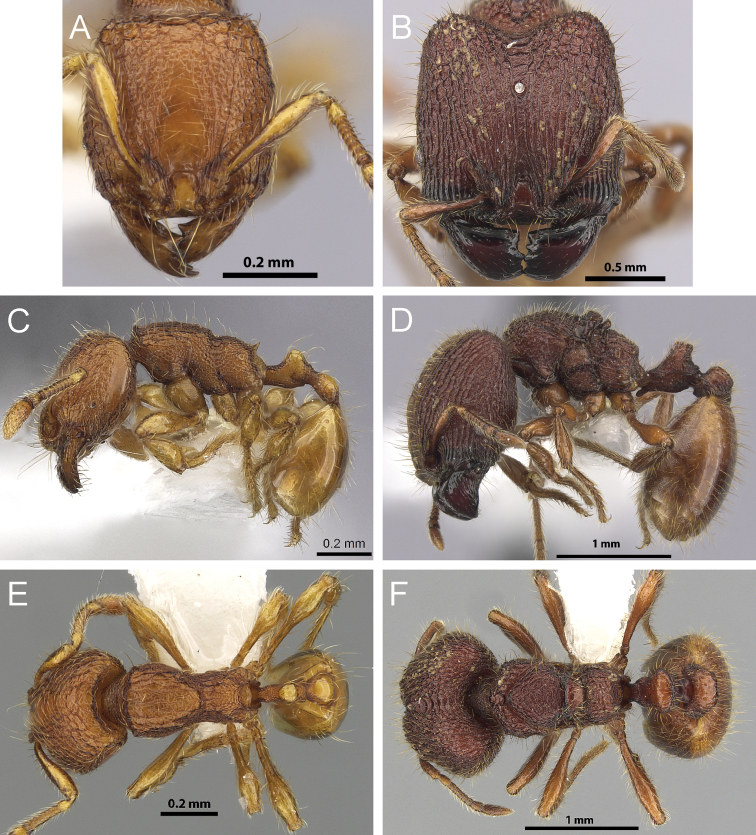
*Carebara urichi*. Minor worker, CASENT0624080: **A** head in full-face view **C** body in profile **E** body in dorsal view. Major worker, CASENT0624077: **B** head in full-face view **D** body in profile **F** body in dorsal view.

#### Description of major workers.

Measurements (n=4): HW 0.97–0.99 (0.98), HL 1.01–1.06 (1.04), SL 0.41–0.44 (0.43), MDL 0.53–0.54 (0.53), EL 0.08, WL 0.97–1.02 (0.99), PNH 0.53–0.57 (0.54), PNW 0.56–0.61 (0.58), MNH 0.64–0.68 (0.66), PDH 0.42–0.46 (0.45), PTL 0.42–0.46 (0.44), PPL 0.25–0.26 (0.26), PTH 0.35–0.38 (0.37), PPH 0.26–0.30 (0.28), PTW 0.31–0.35 (0.33), PPW 0.38–0.43 (0.41), PSL 0.11–0.14 (0.13), MFL 0.58–0.61 (0.60), MTL 0.44–0.46 (0.45), CI 93–95 (94), SI 43–44 (44), MDI 54–55 (55), EI 8–9 (8), FI 60–62 (61), PSLI 12–14 (13), LPpI 88–96 (91), DPpI 152–167 (161), PpWI 124–132 (127), PpLI 57–60 (58), PpHI 74–80 (77).

Head slightly longer than wide (CI 93–95), in full-face view nearly subquadrate. Posterior margin of head with deep, V-shaped emargination, posterolateral corners rounded, sides subparallel and almost straight. Mandibles with five teeth. Anterior margin of clypeus nearly straight, sides convex. Frontal carinae absent to inconspicuous. Antenna with eleven segments, scapes short and reaching to about midlength of head (SI 43–44). Eyes present, multi-faceted, and comparatively large (EI 8–9).

In profile, promesonotum queen-like with moderately high pronotum, comparatively long scutum, and short scutellum, the latter slightly to strongly raised above convex to weakly convex dorsal outline of mesosoma. Promesonotal suture on dorsum present as broad impression, metanotum small and obliquely raised posteriorly. Propodeum higher than long in profile, with dorsal face oblique, declining posteriorly, propodeal spines and posterolateral lamella absent to reduced to slightly raised, posterior corners bluntly angulate, posterior declivity subvertical with well-developed lobe. Propodeal spiracle roundly convex and situated closer to dorsum of propodeum than to its center, not close to posterior declivity.

Petiole with relatively short peduncle, anteroventrally concave, with very conspicuous, short to relatively long, finger-like anterior process, posteroventrally convex, petiole node high, anteriorly oblique to weakly concave, posteriorly vertical and weakly concave, dorsum straight with a rounded angle anteriorly and a sharp right angle posteriorly. Postpetiole in profile squarish with rounded corners and lower than petiole (PpHI 74-80), with shallow, anteriorly angulate ventral process, anterior face almost vertical, dorsum almost straight and posterior face short and oblique. In dorsal view, petiole node much wider than long and anteroposteriorly flattened, anteriorly slightly convex, posterioly transverse and sharply marginate, postpetiole with wide, lamellate processes extending lateroventrally (PpWI 124-132), the node itself narrower than petiole and slightly wider than long, with convex sides, and anterior and posterior margins almost transverse.

Mandibles smooth and shiny, laterally weakly striate and with scattered punctures. Clypeus smooth and shiny, with several weak lateral carinae. Sculpture on frons and anterior sides of head with strong, irregular rugae, posterior parts of face coarsely rugoreticulate, interspaces weakly to superficially punctate, ventral side of head with long, parallel, longitudinal rugae. Mesosoma mostly coarsely and very irregularly rugoreticulate with punctate ground sculpture, rugoreticulate sculpture can be reduced on lateral surfaces, anepisternum sometimes with smooth and shiny area posteriorly. Petiole node and postpetiole dorsally smooth and shiny to faintly punctate, gaster shagreened or superficially punctate. Head and body usually with abundant, erect to suberect hairs of varying length and shorter decumbent to suberect pilosity, scapes and tibiae with appressed to decumbent pilosity and long suberect to erect hairs along outer edge. Color reddish brown, appendages and gaster light brown.

#### Description of minor workers.

Measurements (n=5): HW 0.36–0.44 (0.40), HL 0.41–0.51 (0.46), SL 0.29–0.34 (0.32), MDL 0.23–0.29 (0.26), EL 0.02, WL 0.45–0.59 (0.53), PNH 0.17–0.22 (0.19), PNW 0.23–0.27 (0.25), MNH 0.25–0.31 (0.28), PDH 0.17–0.22 (0.19), PTL 0.15–0.18 (0.16), PPL 0.11–0.12 (0.11), PTH 0.09–0.11 (0.10), PPH 0.08–0.09 (0.08), PTW 0.07–0.08 (0.08), PPW 0.08–0.10 (0.09), PSL 0.07–0.08 (0.07), MFL 0.30–0.39 (0.34), MTL 0.26–0.32 (0.29), CI 86–88 (87), SI 76–91 (81), MDI 62–66 (65), EI 4–6 (5), FI 80–91 (84), PSLI 17–19 (18), LPpI 139–160 (147), DPpI 69–81 (75), PpWI 109–122 (115), PpLI 67–80 (71), PpHI 71–83 (77).

Head almost as wide as long (CI 93–95), posterior margin nearly straight to weakly concave, sides convex. Mandible with four well-defined teeth, apical and preapical tooth larger than others. Anterior margin of clypeus very weakly concave, at each side with a triangular tooth. Frontal carinae moderately long, sometimes reaching posterior third of head. Antenna with eleven segments, scape short and reaching about 6/7 towards posterior margin of head (SI 76–91). Eyes consisting of one ommatidium (EI 4–6).

In profile, dorsum of promesonotum weakly convex, anteriorly sharply marginate and right-angled, posteriorly curving slightly downwards toward widely, but shallowly impressed metanotal groove. Dorsum of propodeum convex, softly declining posteriorly and slightly shorter than posterior declivity, anterodorsal corner convex, propodeal spines relatively short, acute (PSLI 14–15) and lamellate, lamella proceeding ventrally, ending in large propodeal lobes. Propodeal spiracle rounded and situated close to base of spines.

Petiole in profile with peduncle about as long as petiole node, anteroventrally concave, with small, acute, anteriorly pointing ventral process, posteroventrally weakly convex, petiole node low and dorsally rounded. Postpetiole in profile dorsally convex, ventrally almost straight, on average 1.5 times longer than high (LPpI 139–160), lower than petiole (PpHI 71–83). In dorsal view, petiole node almost as wide as long, anteriorly roundly convex, posteriorly nearly straight or weakly convex, postpetiole longer than wide (DPpI 69–81), slightly wider than petiole (PpWI 109–122), anteriorly tapering and posteriorly weakly concave. Gaster slender in dorsal view, its anterior margin straight to faintly convex.

Mandibles, clypeus and center of frons smooth and shiny, remainder of face coarsely rugoreticulate, with scattered punctures. Promesonotum dorsally with several irregular longitudinal rugae, interspaces superficially punctate to smooth, remainder of mesosoma alveolate, alveolae on basal lateropronotum sometimes strongly effaced. Gaster and dorsum of petiole and postpetiole smooth and shiny, remainder of waist segments weakly alveolate.

Head and body with long, suberect to erect hairs and shorter subdecumbent to decumbent pilosity. Scapes and tibiae with decumbent to subdecumbent pilosity, the latter with longer suberect hairs along outer edge. Color orange to dark orange with lighter colored antennae, legs and gaster.

#### Distribution and biology.

*Carebara urichi* is widespread in the Neotropical Region and was found in Belize, Brazil, Colombia, Costa Rica, Mexico, Panama, Peru, Suriname and Trinidad, mainly in rainforest and cloudforest and at elevations ranging from 20–1470 m. Individuals and nest series were collected from the leaf-litter using Winkler sifting.

#### Comments.

*Carebara urichi* can be confused with *Carebara brevipilosa*, but is easily separated by the sculpture on the dorsal promesonotum, which is irregularly longitudinally rugose to rugoreticulate with few irregular longitudinal rugae in *Carebara urichi* minor workers and weakly to superficially reticulate or with few very short rugulae in *Carebara brevipilosa*. *Carebara urichi* and *Carebara brevipilosa* are the only two species in the *Carebara polita* group recorded for the Neotropical Region.

#### Material examined.

**COSTA RICA:** Heredia, La Selva Biol. Sta., 50m, 23.xi.1999 (Project ALAS); Limon, 3 km SSE Cahuita, 70m, 24.xii.1983 (P.S. Ward); **PANAMA:** Barro Colorado, ix.1941 (J.A.S. Zetek).

### Carebara villiersi

Taxon classificationAnimaliaHymenopteraFormicidae

(Bernard)

Figure 17

Nimbamyrma villiersi Bernard, 1953: 241. Syntypes (2 minor workers): GUINEA: Mt Nimba, forest, moss, ix.1946 (*Villiers*). [1 syntype examined, MNHN: EY0000000330/CASENT0913610].
Oligomyrmex villiersi (Bernard): Ettershank 1966: 124 (combination).
Carebara villiersi (Bernard): Fernández 2004: 235 (combination).

#### Diagnosis.

Antenna with eleven segments. **Major workers:** unknown. **Minor worker:** Head shape rounded, sides strongly convex and posterior margin straight to faintly convex medially, face smooth and shiny, frontal carinae short to inconspicuous, dorsal face of propodeum nearly straight or weakly convex, anepisternum and katepisternum areolate, propodeal spines long and spinose, in dorsal view longer than distance between them, gaster with sparse hairs, some hairs moderately long and suberect.

**Figure 17.**
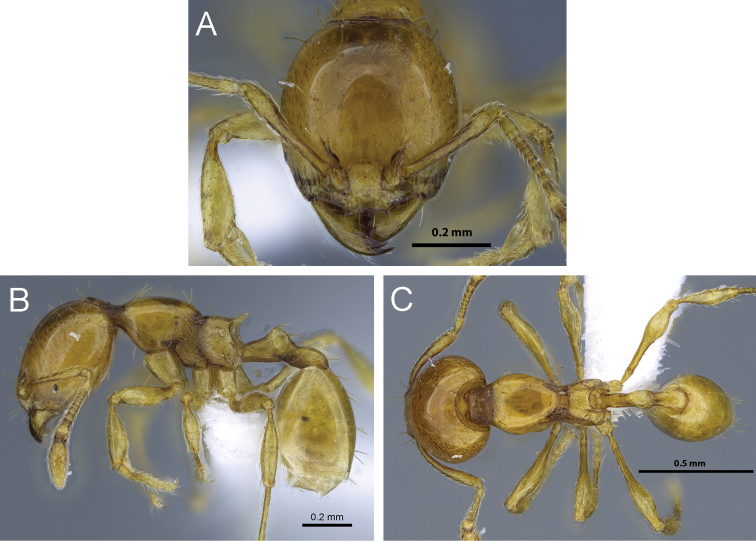
*Carebara villiersi*. Minor worker, CASENT0914220: **A** head in full-face view **B** body in profile **C** body in dorsal view.

#### Description of minor workers.

Measurements (n=5): HW 0.44–0.49 (0.47), HL 0.50–0.54 (0.52), SL 0.37–0.38 (0.38), MDL 0.27–0.29 (0.28), EL 0.02–0.03 (0.03), WL 0.52–0.54 (0.53), PNH 0.20–0.21 (0.21), PNW 0.26–0.28 (0.27), MNH 0.29–0.31 (0.30), PDH 0.20–0.22 (0.21), PTL 0.21–0.23 (0.22), PPL 0.13–0.14 (0.14), PTH 0.14–0.15 (0.14), PPH 0.10–0.11 (0.10), PTW 0.08–0.09 (0.08), PPW 0.10–0.11 (0.10), PSL 0.10–0.11 (0.10), MFL 0.40–0.42 (0.41), MTL 0.30–0.34 (0.31), CI 87–90 (89), SI 78–86 (81), MDI 58–62 (60), EI 5–7 (6), FI 85–93 (88), PSLI 21–23 (22), LPpI 131–146 (138), DPpI 68–81 (75), PpWI 113–130 (123), PpLI 59–68 (63), PpHI 68–72 (70).

Head longer than wide (CI 87–90), in full-face view rounded oval to subcircular, narrowed anteriorly and posteriorly, posterior margin weakly concave. Sides strongly convex, smoothly rounding into posterolateral corners. Mandible triangular with five teeth apical and preapical tooth larger than the others. Anterior margin of clypeus concave, bicarinate and divergent forward, sides with short triangular teeth. Antennae with eleven segments, scape almost reaching posterior margin of head (SI 78–86). Eyes consisting of one ommatidium, situated comparatively close to anterolateral margin of head (EI 5–7).

In profile, promesonotum convex, metanotal groove moderately impressed. Dorsum of propodeum short in profile and slightly convex, posteriorly with long, acute, blade-like, and posteriorly pointing spines, in dorsal view distinctly longer than distance between their bases, declivity of propodeum faintly concave to straight and oblique, with straight lamella. Propodeal spiracle rounded and situated near the base of the spines.

Petiole in profile with peduncle slightly longer than petiole node, ventrally almost straight with straight and shallowly lamellate anterior process and rounded posterior corner, petiole node low, anterodorsally and posterodorsally with short, weakly concave faces, dorsal face almost straight and subangulate anteriorly and posteriorly. Postpetiole in profile on average 1.4 times longer than high (LPpI 131–146), lower than petiole (PpHI 68–72), anteroventrally weakly convex. In dorsal view, petiole node narrow, distinctly longer than wide, anteriorly tapering, posteriorly almost straight, postpetiole distinctly longer than wide (DPpI 68–81), slightly wider than petiole (PpWI 113–130), anteriorly tapering, almost pedunculate, and posterior face convex. Gaster anterolaterally with acute shoulders around posterior base of postpetiole.

Mandibles and head smooth and shiny with some punctures, except on frons, malar region anterior of eye level with short rugulae. Mesosoma and metasoma mostly smooth and shiny, except for weak areolae on mesopleuron and metapleuron, comparatively long cross-ribs at dorsal metanotal groove, superficial areolae on anterolateral border of propodeum and on posterolateral lamella. Anterodorsal and ventral surface of petiole weakly to superficially areolate, ventral face of postpetiole weakly punctate.

Standing hairs relatively short and stout, not abundant, evenly distributed, and mostly arrayed in pairs from head to gaster. Scapes and metatibiae with appressed to weakly decumbent pilosity. Color yellowish orange.

#### Distribution and biology.

*Carebara villiersi* was described from the Nimba Mountains in Guinea and can also be found in Cameroon, Central African Republic, Gabon, Ghana, and Ivory Coast, where it was collected in rainforest at elevations ranging from 20–1470 m. Individuals and nest series were collected from leaf-litter using Winkler sifting.

**Figure 18.**
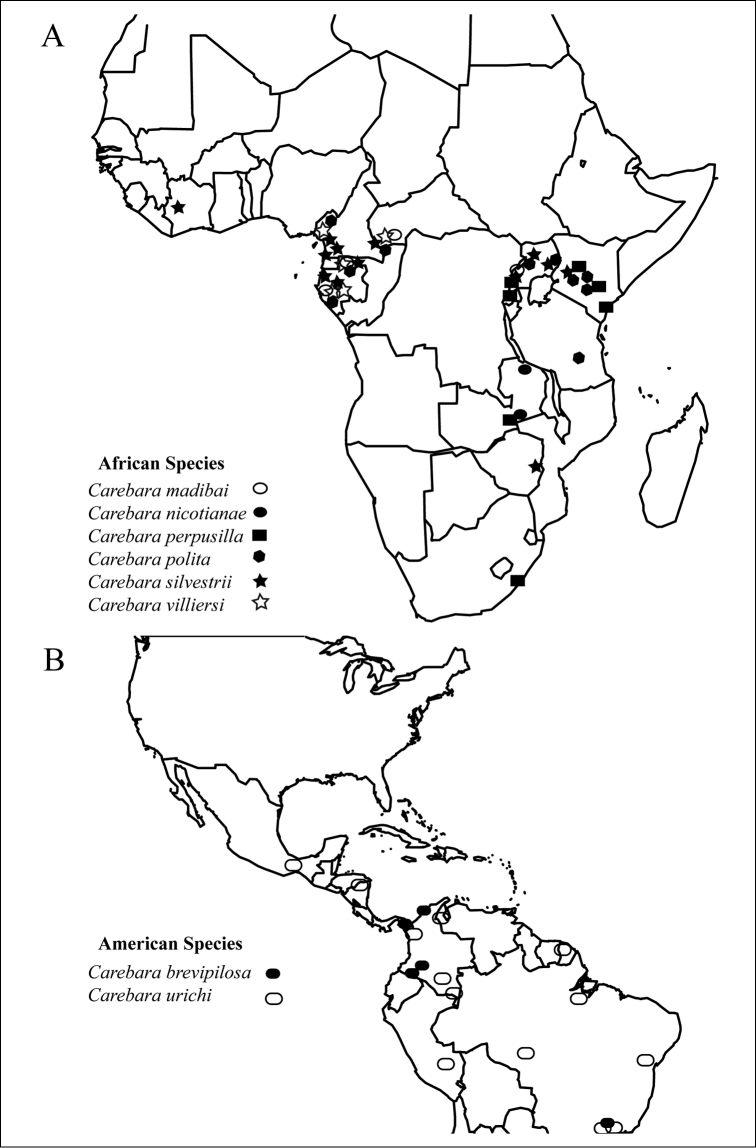
Distribution maps for the *Carebara polita* group: **A** African species **B** American species.

#### Comments.

Minor workers of *Carebara villiersi* can be confused with *Carebara nicotianae* and *Carebara polita*, but can be easily separated from them, because the mesopleuron and metapleuron are areolate in *Carebara nicotianae* and smooth and shiny in *Carebara polita*, but the mesopleuron is areolate and the metapleuron smooth and shiny in *Carebara villiersi*. Major workers either not existing or not yet collected or identified as such.

#### Material examined.

**CAMEROON:** Sud-Ouest, Korup N. P., 6.9 km 317° NW Mundemba, 5.016, 8.864, 110m, rainforest, 19.iv.2000 (*B.L. Fisher*); **CENTRAL AFRICAN REPUBLIC:** Prefecture Sangha-Mbaéré, Parc National Dzanga-Ndoki, 38.6 km 173° S Lidjombo, 2.36, 16.14397, 350m, rainforest, 21–27.v.2001 (*S. Van Noort*); **GABON:** Ogooue-Maritime, Reserve de Faune de la Moukalaba Dougoua, 12.2 km 305° NW Doussala, -2.31667, 10.53333, 110m, rainforest, 24.ii.2000 (*B.L. Fisher*); Ogooue-Maritime, Aire d'Exploit. Rationnelle de Faune des Monts Doudou, 24.5 km 303° WNW Doussala, -2.23283, 10.398, 630m, rainforest, 18.iii.2000 (*B.L. Fisher*); Woleu-Ntem, 31.3 km 108° ESE Minvoul, 2.08, 12.40667, 600m, rainforest, 7.ii.1998 (*B.L. Fisher*).

## Supplementary Material

XML Treatment for Carebara brevipilosa

XML Treatment for Carebara madibai

XML Treatment for Carebara nicotianae

XML Treatment for Carebara perpusilla

XML Treatment for Carebara polita

XML Treatment for Carebara silvestrii

XML Treatment for Carebara urichi

XML Treatment for Carebara villiersi
